# Targeting pyruvate metabolism generates distinct CD8^+^ T cell responses to gammaherpesvirus and B lymphoma

**DOI:** 10.1172/jci.insight.187680

**Published:** 2025-08-22

**Authors:** Taewook Kang, Young-Kwang Usherwood, Julie A. Reisz, Sukrut C. Kamerkar, Rachel Culp-Hill, Owen M. Wilkins, Andreia F. Verissimo, Fred W. Kolling, Anton M. Hung, Shawn C. Musial, Pamela C. Rosato, Angelo D’Alessandro, Henry N. Higgs, Edward J. Usherwood

**Affiliations:** 1 Microbiology and Immunology Department, Geisel School of Medicine, Dartmouth College, Lebanon, New Hampshire, USA.; 2 Metabolomics core, University of Colorado Anschutz Medical Campus, Aurora, Colorado, USA.; 3 Department of Biochemistry and Cell Biology, Geisel School of Medicine, Dartmouth College, Hanover, New Hampshire, USA.; 4 Genomics and Molecular Biology Shared Resource, Dartmouth Cancer Center, Lebanon, New Hampshire, USA.; 5 Department of Biomedical Data Science, Geisel School of Medicine, and; 6 Institute for Molecular Targeting, Geisel School of Medicine, Dartmouth College, Hanover, New Hampshire, USA.

**Keywords:** Immunology, Metabolism, Adaptive immunity, Immunotherapy

## Abstract

T cells rely on different metabolic pathways to differentiate into effector or memory cells, and metabolic intervention is a promising strategy to optimize T cell function for immunotherapy. Pyruvate dehydrogenase (PDH) is a nexus between glycolytic and mitochondrial metabolism, regulating pyruvate conversion to either lactate or acetyl-CoA. Here, we retrovirally transduced pyruvate dehydrogenase kinase 1 (PDK1) or pyruvate dehydrogenase phosphatase 1 (PDP1), which control PDH activity, into CD8^+^ T cells to test effects on T cell function. Although PDK1 and PDP1 were expected to influence PDH in opposing directions, by several criteria they induced similar changes relative to control T cells. Seahorse metabolic flux assays showed both groups exhibited increased glycolysis and oxidative phosphorylation. Both groups had improved primary and memory recall responses following infection with murine gammaherpesvirus-68. However, metabolomics using labeled fuels indicated differential usage of key fuels by metabolic pathways. Importantly, CD8^+^ T cell populations after B cell lymphoma challenge were smaller in both groups, resulting in poorer protection, which was rescued by glutamine and acetate supplementation. Overall, this study indicates that PDK1 and PDP1 both enhance metabolic capacity, but the context of the antigenic challenge significantly influences the consequences for T cell function.

## Abstract 2

Enforced expression of enzymes regulating the activity of pyruvate dehydrogenase kinase change the differentiation and functional state of CD8 T cells in a context-dependent manner

## Graphical abstract

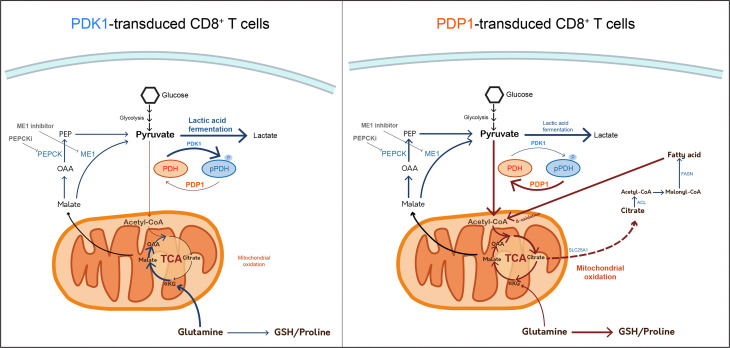

## Introduction

CD8^+^ T cells are immune cells that confer protection against different pathogens or tumors throughout the body and are actively being used for mediating immunotherapy. Chimeric antigen receptor T (CAR-T) cell therapy, an example of adoptive T cell therapy, has shown promising efficacy against cancer, especially blood cancers, and holds promise for viruses and autoimmune diseases (1–4). However, eliciting effective T cell–mediated immunity and long-lasting memory T cell responses in adoptive therapy remains challenging (5, 6). Thus, understanding how the longevity and potency of T cells can be enhanced is critical in improving T cell immunotherapy.

T cells undergo activation and differentiation in various tissues and antigenic environments, and this energetically demanding process depends on nutrients and metabolites (7). By supporting cell proliferation and controlling function, metabolic reprogramming influences how T cells adapt to the surrounding antigenic context in tissue environments and changes the functionality and persistence of T cells (8). A striking example of an immune microenvironment affecting T cell metabolism is in cancer. The tumor microenvironment (TME) inhibits the metabolic adaptation of T cells by nutrient depletion, production of immunosuppressive metabolites, and reduced mitochondrial fitness leading to T cell exhaustion and functional defects (9). CD8^+^ T cell metabolic programming leads to changes in their function and differentiation also during viral or intracellular bacterial infections (10–16). CD8^+^ T cells, for example, during influenza infection without CD4^+^ T cell help are metabolically dysfunctional and exhibit an exhausted phenotype (17). These metabolic challenges T cells face in the context of cancer and infections underline the importance of appropriate metabolic programming to support a protective T cell response.

Within cellular metabolism, glycolysis rapidly breaks down glucose providing intermediates for other metabolic pathways, while mitochondrial oxidative phosphorylation (OXPHOS) generates ATP molecules efficiently through the TCA cycle (18). T cells with tight mitochondrial cristae and enhanced OXPHOS can form long-lived memory cells with reduced mitochondrial reactive oxygen species (ROS) and improved antitumor efficacy (19). Studies that intentionally skewed metabolism toward OXPHOS and away from glycolysis have shown evidence of improved persistence and T cell activity in autoimmune, cancer, and infection settings (11, 20, 21). In contrast, constitutively glycolytic CD8^+^ T cells with deletion of Vhl, which degrades HIFα, a key positive regulator of glycolysis, promoted effector T cell differentiation, which nevertheless persisted long-term as effector memory T cells against chronic infection (22). These studies, and others, highlight the necessity of understanding how the balance of glycolysis and OXPHOS affects memory T cell differentiation, particularly in immunologically challenging environments, such as the presence of persistent antigens.

Pyruvate, as a product of glycolysis, can be fermented to lactate, but it can also be oxidized further in the TCA cycle after being converted into acetyl-CoA by the pyruvate dehydrogenase (PDH) complex. This pathway selection is regulated by PDH E1α phosphorylation, which inactivates the complex and drives lactic acid conversion. Pyruvate dehydrogenase kinase 1 (PDK1) and pyruvate dehydrogenase phosphatase 1 (PDP1) are key regulatory isoenzymes that modify the phosphorylation of PDH E1α, which can drive metabolic reprogramming in T cells (23–25). All 3 residues of Ser^232^, Ser^293^, and Ser^300^ of PDH E1α can be phosphorylated and dephosphorylated by PDK1 and PDP1, respectively (26). Therefore, the engineering of PDH phosphorylation regulators can control fuel utilization in the mitochondria and T cell differentiation. Previous studies have discovered a role for PDK1 in enhancing glycolysis after T cell activation, and active PDH can limit Th17 differentiation (23, 24, 27). However, it is less well understood how the balance between mutually antagonizing PDK and PDP changes CD8^+^ T cells and their resulting differentiation and function during different antigenic challenges.

We hypothesized that enforced *Pdk1* expression would drive CD8^+^ T cells toward glycolytic metabolism while enforced *Pdp1* expression would promote mitochondrial metabolism in the T cells leading to different effector and memory T cell differentiation. In this study, we used animal models of persistent murine gammaherpesvirus-68 (MHV-68) infection and EμMyc B cell lymphoma physiologically relevant to this virus (28) to understand how changes in PDH activity affected memory and effector CD8^+^ T cell differentiation. Our data suggest that retrovirally transduced PDK1 and PDP1 induced higher metabolic capacity in CD8^+^ T cells with larger primary and secondary responses against MHV-68, while they induced poor protection against B cell lymphoma challenge compared with the control T cells, potentially due to the altered glutamine and fatty acid within the TME. These data provide insights into how metabolic targeting of CD8^+^ T cells can result in different outcomes based on the nature of the immunological challenge.

## Results

### Retroviral transduction of PDH phosphoregulatory enzymes changes PDH phosphorylation.

**Figure 1:**
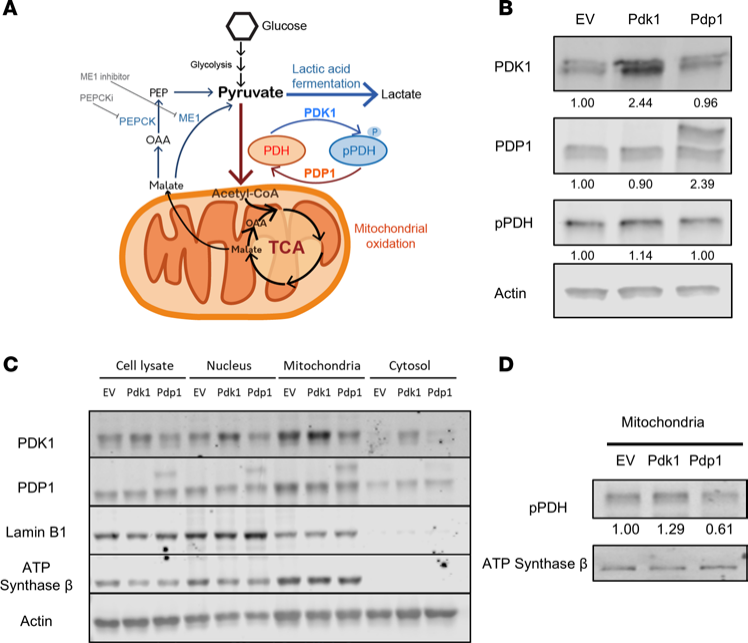
Retrovirally introduced *Pdk1* or *Pdp1* changes mitochondrial PDH phosphorylation status in CD8^+^ T cells. (**A**) Schematic summary of PDH phosphorylation regulation by PDK1 and PDP1 controlling carbon utilization in the mitochondrial TCA cycle. (**B**) Protein levels in mouse CD8^+^ T cells were analyzed by Western blot with retrovirally transduced T cells expressing EV, *Pdk1*, or *Pdp1*. Cells harvested 5 days after transduction were lysed for protein detection with anti-PDK1, PDP1, phosphorylated PDH (pSer300), or actin antibodies. Numbers indicate relative abundance of each protein normalized relative to actin. (**C**) Western blot data with the transduced protein expression in subcellular organelle fractions enriched by differential centrifugation. Lamin B1, ATP synthase β, and actin are markers for nuclear, mitochondrial, and cytosolic fractions, respectively. (**D**) The phosphorylation status of PDH was affected by enforced PDK1 and PDP1 expression in enriched mitochondrial fractions. Each experiment was repeated 2 to 3 times.

As the PDH complex controls the bifurcation of glycolysis and OXPHOS, we hypothesized that manipulating PDH complex activity would change the differentiation and function of CD8^+^ T cells (Figure 1A). We transduced mouse CD8^+^ T cells with the pCI-CD19 retroviral vector encoding *Pdk1* or *Pdp1* after anti-CD3/CD28 stimulation in vitro. Following positive selection for the reporter CD19 extracellular domain, the purity of the retrovirally transduced cells was higher than 97% (Supplemental Figure 1; supplemental material available online with this article; https://doi.org/10.1172/jci.insight.187680DS1).

First, we verified protein expression after transduction of mouse CD8^+^ T cells with *Pdk1*- or *Pdp1*-encoding retroviruses. From whole CD8^+^ T cell lysate, the overexpression of PDK1 protein was verified in *Pdk1*-transduced cells compared with the empty vector (EV) control (Figure 1B and Supplemental Figure 2A). PDP1 protein with a heavier molecular weight was detected only in the *Pdp1*-transduced cells. This is consistent with the variant 4 isoform selected for expression in the retroviral vector, which has a longer N-terminal region. We also checked the phosphorylation of PDH to confirm the kinase and phosphatase activity on the target substrate PDH. PDH E1α phosphorylated (p-PDH) on Ser^300^ (Figure 1B) was not markedly changed in the whole-cell lysates. Thus, we also measured the presence of the transduced proteins in mitochondria and other subcellular compartments. Nuclear, mitochondrial, and cytosolic fractions were isolated by differential centrifugation (29). Lamin B1, ATP synthase β, and actin proteins were used as internal controls for nuclear, mitochondrial, and cytosolic fractions, respectively. The transduced PDK1 and PDP1 proteins were more abundant in the relevant transduced groups than in EV controls (Figure 1C and Supplemental Figure 2, B and C). As expected, PDK1 and PDP1 were expressed most prominently in the mitochondrial fraction. However, they were also present in the nucleus, consistent with known nuclear PDH activity generating acetyl-CoA for histone acetylation (30–32). Both enzymes were also detected at lower levels in the cytosol (Figure 1C). In contrast with the whole-lysate result, more mitochondrial PDH E1α Ser^300^ phosphorylation was observed with *Pdk1* expression, consistent with expectations. Meanwhile, phosphorylation was reduced with *Pdp1* expression, relative to controls (Figure 1D and Supplemental Figure 2D). Stable expression of transduced proteins was verified for at least 20 days in vitro (Supplemental Figure 2, E and F). These results showed that retroviral transduction of Pdk1 and Pdp1 led to higher protein expression levels in the mitochondria and changed the phosphorylation status of PDH E1α.

### Manipulation of PDH activity enhances CD8^+^ T cell metabolism in vitro.

**Figure 2:**
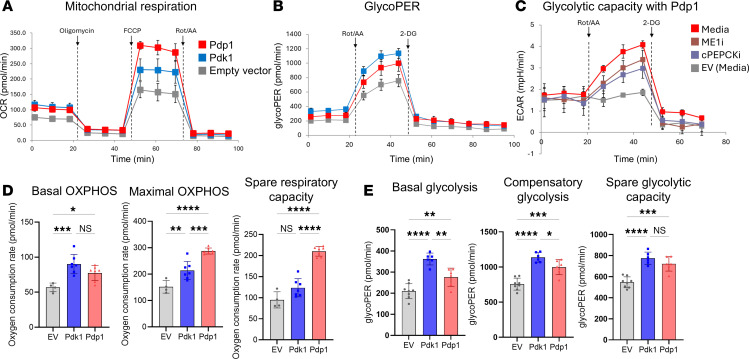
Enforced PDK1 or PDP1 expression induces higher metabolic capacity in CD8^+^ T cells. Metabolic profiles of CD8^+^ T cells expressing PDK1 and PDP1 were analyzed by Seahorse assay. Cells were cultured in vitro with IL-2 for 3 days and were analyzed for (**A**) oxygen consumption rate with mitostress assay for mitochondrial respiratory capacity and (**B**) glycoPER with glycolytic rate assay for glycolytic capacity. (**C**) Pdp1-transduced cells were incubated with the inhibitors of ME1 or cPEPCK for 1.5 hours before analysis by glycolytic rate assay with media control– and EV-transduced cells. (**D**) Basal, maximal OXPHOS, and spare respiratory capacity from **A**. (**E**) Basal, compensatory, and spare glycolytic capacity levels from **B**. Each experiment was repeated 3 times. *n* = 4–8 technical replicates per group; 1-way ANOVA corrected for multiple comparisons with Tukey’s multiple comparisons test. *P < 0.05; **P < 0.01; ***P < 0.001;****P < 0.0001. Data are mean ± SD. OCR, oxygen consumption rate; ECAR, extracellular acidification rate; ME1, malic enzyme 1; cPEPCK, cytosolic phosphoenolpyruvate carboxykinase; glycoPER, glycolytic proton efflux rate.

To analyze the effect of enforced PDK1 or PDP1 expression on glycolytic metabolism and OXPHOS, we measured the OXPHOS and glycolytic capacity in PDK1- and PDP1-overexpressing cells by the Seahorse glycolytic rate and mitostress assays. Surprisingly, when we transduced CD8^+^ T cells with these 2 genes, both of them showed enhanced mitochondrial respiration and glycolytic rate when compared with the EV control (Figure 2, A–E). In the mitostress assay, the PDP1 group had the highest OCR, especially the maximal level of OXPHOS, whereas the PDK1 group showed a lower maximal OCR level than PDP1 but was significantly higher than the EV control (Figure 2, A and D). The spare respiratory capacity (Max-Min OXPHOS) was significantly increased in the PDP1 group but not in the PDK1 group. Compared with EV controls the highest levels of basal and maximal glycolysis were observed in the PDK1 group. However, the PDP1 group was significantly higher than controls for these parameters. Both groups had significantly elevated spare glycolytic capacity (Max-Min glycolysis) compared with the control (Figure 2, B and E). These data showed that enforced expression of PDH phosphoregulatory enzymes increased both mitochondrial and glycolytic capacity in CD8^+^ T cells.

### Carbon export from the mitochondria fuels lactate production.

These findings were unexpected from our hypothesis wherein PDK1 was expected to skew the cells toward higher glycolysis and lower OXPHOS, whereas PDP1 would display the inverse phenotype. Regarding carbon source utilization, it is increasingly reported that the intermediate molecules of glycolysis and TCA cycle are not solely committed to one pathway but can be transferred in and out of mitochondria to be used in other pathways, such as anaplerosis/cataplerosis and alternative TCA cycle (33–37). TCA intermediate molecules malate and oxaloacetate can be exported to the cytosol and used for regenerating pyruvate by ME1 (38, 39) or gluconeogenesis or pyruvate conversion from phosphoenolpyruvate (PEP) by cPEPCK (or PCK1) (40, 41). As these 2 enzymes can bridge the mitochondrial TCA cycle and cytosolic pyruvate, we thought the unexpected increase in lactic acid production with increased PDH activity could have been derived from TCA cycle intermediates being exported to the cytosol, then converted first to PEP and then lactate, followed by export from the cell (Figure 1A). Thus, we measured the ECAR (lactate export) of CD8^+^ T cells after treatment with inhibitors of ME1 or cytosolic PEPCK using cells transduced with PDP1. Both ME1 or PEPCK inhibitor treatments resulted in reductions in lactate production, measured by ECAR levels (Figure 2C). This was consistent with roles for ME1 and PEPCK in the process of converting TCA cycle intermediates ultimately to lactate. These data support the model that a proportion of carbons preferentially routed to the mitochondria by enforced PDP1 expression subsequently exit the mitochondria and reenter the cytoplasm, where they are converted to pyruvate, then to lactate, followed by export from the cell.

Overall, these results suggest that the transduction of PDK1 and PDP1 induced higher metabolic capacity, indicated by OXPHOS and glycolytic rate, with the export of TCA intermediates contributing to conversion into lactate and higher ECAR readings.

### Transcriptional profiles of CD8^+^ T cells with enforced Pdk1 or Pdp1 expression.

**Figure 3:**
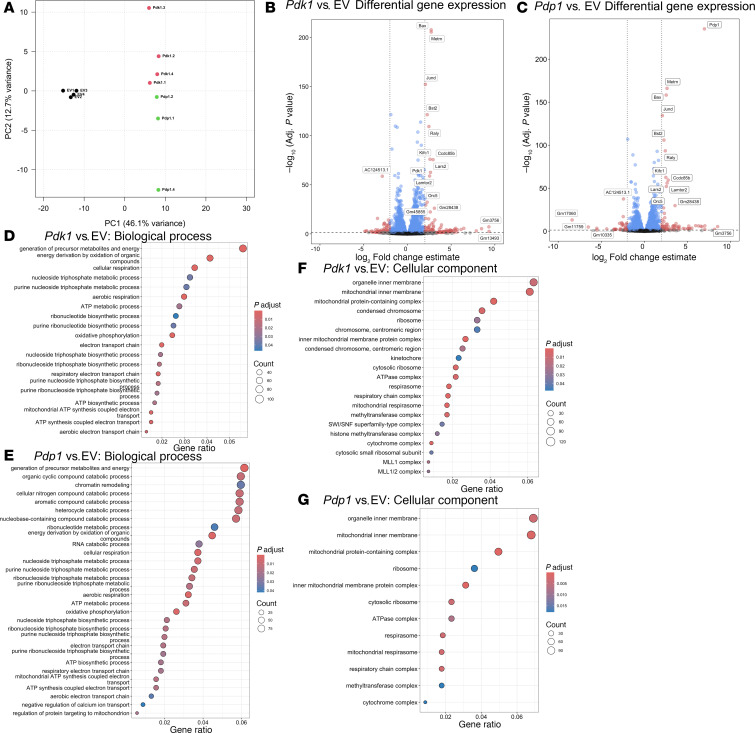
RNA transcription profiles were similar between *Pdk1-* and *Pdp1-*transduced cells. Mouse CD8^+^ T cells retrovirally transduced with EV, Pdk1, or Pdp1 were cultured with IL-2 for 4 days and analyzed using bulk RNA sequencing. (**A**) Principal component analysis plots of EV, *Pdk1*, and *Pdp1* groups. (**B** and **C**) Volcano plots showing the differentially expressed genes (DEGs) for (**B**) *Pdk1* versus EV and (**C**) *Pdp1* versus EV. (**D**–**G**) Functionally enriched pathways from overrepresentation (ORA) analysis of curated GO set collections of Biological Process pathways and Cellular Component pathways. *Pdk1* and *Pdp1* were individually compared with EV. DEGs with adjusted *P* value under 0.05 and fold-change over 0.5 were analyzed. Per group 3 or 4 technical replicate samples were analyzed.

We next compared the transcriptional landscape of 3 groups of transduced CD8^+^ T cells, *Pdk1*, *Pdp1*, and EV, to understand how the enforced expression of PDH phosphorylation regulators affected CD8^+^ T cell transcription. The transduced cells were cultured with IL-2 and then analyzed by bulk RNA sequencing. Statistically significant differentially expressed genes (DEGs) were analyzed to compare EV versus *Pdk1*, EV versus *Pdp1*, and *Pdk1* versus *Pdp1*. Principal component (PC) analysis showed consistent clustering of replicates among groups (Figure 3A). PC1 separated EV samples from *Pdk1*/*Pdp1* samples and explained 46.1% of the variance while *Pdk1* and *Pdp1* were separated by PC2 with 12.7% of variance explained, suggesting differences in expression between *Pdk1*- and *Pdp1*-transduced cells were relatively small in comparison to EV. Inspection of the significant DEGs with the largest fold-changes confirmed the upregulation of transduced genes, *Pdk1* or *Pdp1*, validating effective expression of these genes in transduced T cells (Figure 3, B and C). Interestingly, *Pdk1* and *Pdp1* groups both shared increased expression of *Bax*, *Bst2*, *Jund*, *Raly*, *Ccdc85b*, and *Lamtor2*. JunD is an activator protein 1 (AP-1) family protein controlling T cell differentiation and is known to regulate cytokine production and proliferation (42, 43). *Raly* is an immune response and inflammatory response gene upregulated in both groups (44). It encodes an RNA-binding protein that regulates alternative splicing of AP-1 transcription factors FOS and FOSB. Bax and BST-2 proteins are also involved in T cell proliferation and effector function (45, 46). While *Pdk1* and *Pdp1* groups showed a significant increase in these genes related to cell proliferation and survival compared with EV, few significant DEGs were identified between *Pdk1* and *Pdp1* (Supplemental Figure 3A).

To functionally annotate sets of DEGs from each comparison, we performed an overrepresentation analysis (ORA) using a variety of gene set collections (Gene Ontology [GO] Biological Process and Cellular Component). Enriched gene sets from the Biological Process collection again demonstrated similarity between *Pdk1* and *Pdp1* groups compared with EV (Figure 3, D and E, and Supplemental Data 1). *Pdk1* induced 20 pathways, which could be separated into 2 highly overlapping groups of electron transport chain/ATP synthesis and OXPHOS pathways (Figure 3D and Supplemental Figure 3B). Similarly, *Pdp1* induced 30 pathways clustered into OXPHOS, nucleotide metabolism, and nucleobase/nitrogen compound catabolism (Figure 3E and Supplemental Figure 3C). In Cellular Component GO gene sets, 21 (*Pdk1*) and 12 (*Pdp1*) gene sets related to mitochondrial membrane structure and mitochondrial respiratory chain complexes were enriched in both *Pdk1* and *Pdp1* groups (Figure 3, F and G). These results suggest that the 2 genes opposing PDH phosphorylation state indeed change CD8^+^ T cell metabolism on a transcriptional level in a similar way, consistent with the metabolic phenotypes detected with the Seahorse assay (Figure 2). Importantly, major differences between control and *Pdk1* or *Pdp1* groups were limited to metabolic processes, indicating that enforced expression of these genes does not have effects on other cellular processes beyond modulating cellular metabolism.

### Glucose and lactate tracing show less profound changes in metabolite labeling.

As both PDK1 and PDP1 groups exhibited similar changes in glycolysis and mitochondrial metabolism measured by both transcriptional and functional assays, we next tested whether there was differential usage of key metabolic fuels between these groups.

As our manipulations affected pyruvate metabolism, and the major sources of pyruvate are glucose and lactate, we also tested whether labeling of ^13^C glucose or lactate induced different levels of ^13^C-labeled metabolites. Surprisingly, we did not observe consistent changes in the labeling of glycolytic or TCA cycle intermediates in either PDK1 or PDP1 groups (Supplemental Figure 4). In the glycolysis pathway, fructose-1,6-bisphosphate labeling was increased with PDP1 transduction at 6 hours, but the downstream 3-phosphoglycerate labeling was reduced in PDK1 and PDP1 groups (Supplemental Figure 4A). This result is different from the Seahorse assay result (Figure 2), where the glucose-dependent metabolic capacity was enhanced in both groups. We postulate that this is due to the different nutrient sources present in the 2 assays. This glucose carbon-tracing result suggests that there were no consistent changes in the way cells utilize glucose in the TCA cycle (Supplemental Figure 4B) and glycolysis.

Another metabolite important for T cell differentiation in the nutrient microenvironment is lactate. Although lactate present in the microenvironment is known to be immunosuppressive, it can also be used as a fuel source that contributes to the TCA cycle (47–51). Therefore, we examined metabolite labeling after transduced CD8^+^ T cells were incubated with ^13^C sodium lactate for 1 or 6 hours. The levels of ^13^C_3_ lactate and ^13^C_3_ pyruvate, which indicate the lactate uptake and pyruvate conversion rate, were not significantly different, though lactate uptake was slightly elevated in the PDP1 group (Supplemental Figure 5, A and B). ^13^C labeling in citrate was modestly lower in PDK1 relative to the control, while it was modestly higher in PDP1-transduced cells compared with the control group (Supplemental Figure 5, C and D). There were no statistically significant differences in labeling of other TCA cycle or glycolysis pathway metabolites (Supplemental Figure 5, E–G). Together, the glucose- and lactate-labeling experiments imply that utilization of these 2 nutrients was not strikingly different based on PDK1 or PDP1 transduction, except that lactate-derived citrate accumulated less in the PDK1 group.

### Glutamine-derived TCA cycle intermediates, glutathione, and proline are differentially enriched with PDK1 and PDP1 transduction.

**Figure 4:**
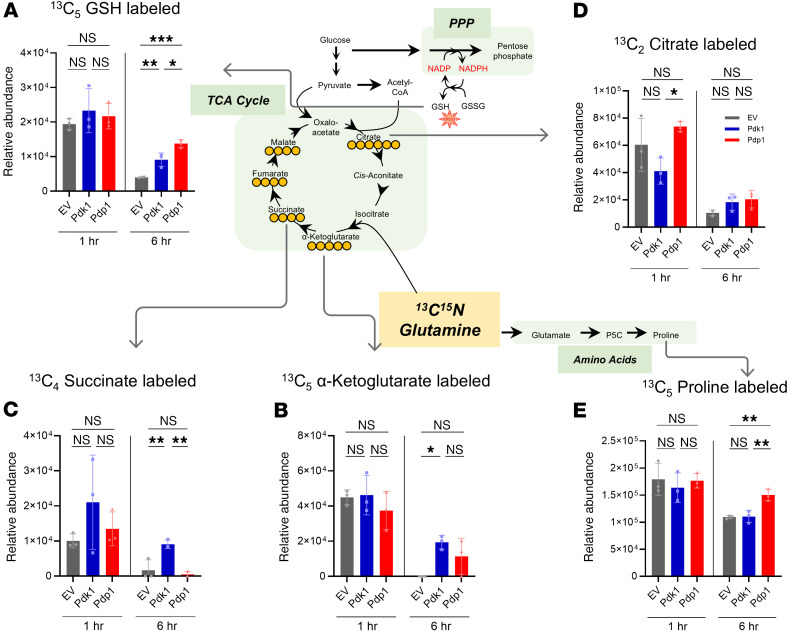
^13^C^15^N glutamine tracing reveals glutamine-derived TCA cycle intermediates, GSH, and proline were differentially enriched in PDK1 or PDP1 groups. CD8^+^ T cells transduced with EV, PDK1, or PDP1 were incubated with ^13^C^15^N glutamine and analyzed with ultrahigh-performance liquid chromatography–mass spectrometry (UHPLC-MS) for metabolite quantification. Detected metabolites showing significant statistical differences (*P* < 0.05) included M+2 citrate, M+5 α-ketoglutarate, M+4 succinate, M+5 GSH, and M+5 proline. Labeled metabolite levels of (**A**) ^13^C_5_ GSH, (**B**) ^13^C_5_ α-ketoglutarate, (**C**) ^13^C_4_ succinate, (**D**) ^13^C_2_ citrate, and (**E**) ^13^C_5_ proline are shown as bar graphs. Groups not compared indicate no statistical significance. *n* = 3 technical replicates per group; 1-way ANOVA corrected for multiple comparisons with Tukey’s multiple comparisons test. *P < 0.05; **P < 0.01; ***P < 0.001. Data are mean ± SD.

Among the nutrients and carbon sources, glutamine is the most abundant nonessential amino acid in the body and is important in T cell activation and metabolic programming (52, 53). Thus, we cultured cells with ^13^C_5_^15^N_2_ glutamine for carbon and nitrogen tracing to track the utilization of glutamine-derived carbon and nitrogen in CD8^+^ T cells. There was no difference in the uptake of labeled glutamine between experimental groups (Supplemental Figure 6A and Supplemental Data 2). Similarly, comparable levels of labeled glutamate were present in all groups, indicating similar glutaminase activity (Supplemental Figure 6B). Interestingly, labeled antioxidant ^13^C_5_ glutathione (GSH) synthesized from glutamate was enriched in both groups, especially in PDP1, after 6 hours (Figure 4A). Similar increases in GSH accumulation with different isotopologs were also observed with the total labeled GSH after 6 hours (Supplemental Figure 6C).

Among TCA cycle intermediates after the entry of glutamine-derived α-ketoglutarate into the TCA cycle, ^13^C_5_ α-ketoglutarate and ^13^C_4_ succinate were increased in PDK1 relative to EV, which was most apparent after 6 hours (Figure 4, B and C). Similar differences with the same trend were also observed in malate and fumarate, but the differences were not significant (Supplemental Figure 6, D and E). Similar to the lactate-labeling results, ^13^C_2_ citrate was reduced in PDK1 compared with EV and PDP1 after 1 hour of incubation, but this was marginally higher in both PDK1 and PDP1 after 6 hours (Figure 4D). Similar trends of ^13^C incorporation were observed in ^13^C_3_ and ^13^C_4_ citrate (Supplemental Figure 6, F and G). This suggests that glutamine-derived carbons were replenishing TCA cycle intermediates, which can generate NADH and FADH_2_ to a greater extent in the PDK1 group (54).

^13^C glutamine-derived ^13^C_5_ proline was enriched in the PDP1 group at 6 hours (Figure 4E). A similar difference was also observed in the total labeled prolines of different isotopologs (Supplemental Figure 6H). The altered utilization of glutamine-derived carbons correlated with the hypothesized changes in pyruvate-derived TCA intermediates with PDK1 and PDP1 transduction. Since PDK1 suppresses the pyruvate-derived acetyl-CoA incorporation into the TCA cycle, glutamine likely supplements the supply of TCA cycle intermediates. The PDP1 group, on the other hand, may utilize glutamine to generate other metabolites, such as proline and GSH, as the cells have sufficient TCA intermediates to maintain the cycle. These results demonstrate that PDK1 increases the carbon flux from glutamine to the TCA cycle while PDP1 also shows the accumulation in proline and GSH.

### Palmitate oxidation and incorporation into TCA cycle intermediates were altered after PDK1 and PDP1 transduction.

**Figure 5:**
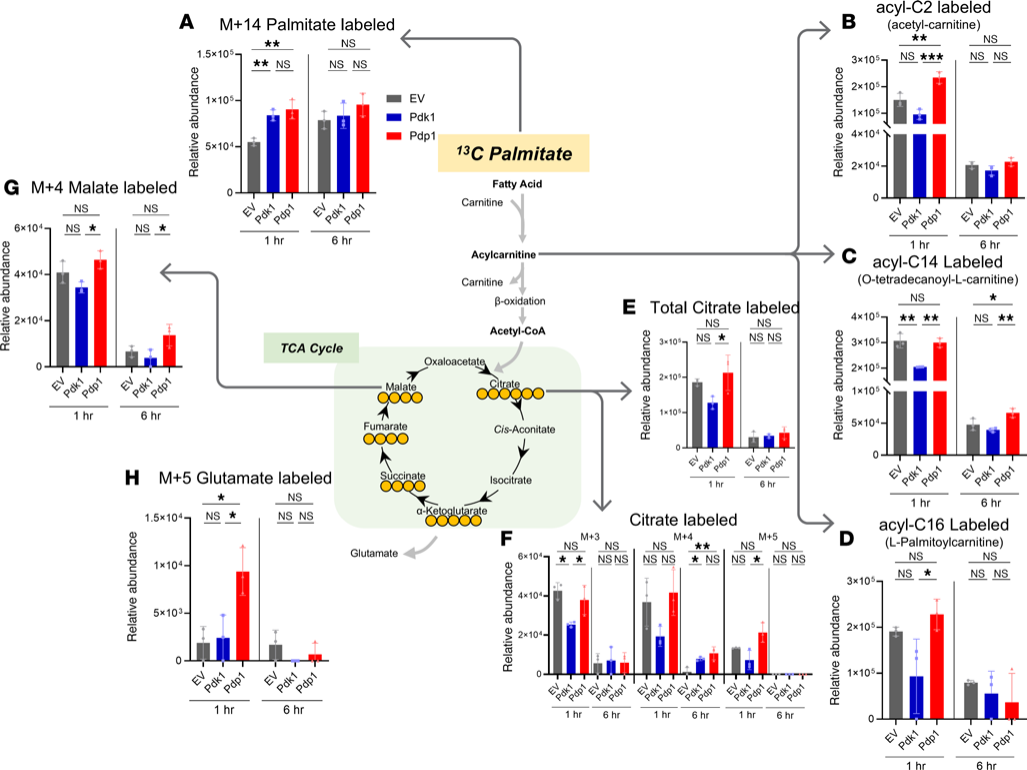
^13^C palmitate tracing reveals altered fatty acid breakdown and TCA cycle incorporation in PDK1 or PDP1 groups. CD8^+^ T cells transduced with EV, PDK1, or PDP1 were incubated with ^13^C palmitate and analyzed with UHPLC-MS for metabolite quantification. Detected metabolites showing significant statistical differences (*P* < 0.05) included (**A**) M+14 palmitate and (**B**–**D**) acyl-carnitines of different lengths. (**E** and **F**) Isotopologs of citrate with different numbers of ^13^C, (**G**) M+4 malate, and (**H**) M+5 glutamate. Relative metabolite levels of these labeled isotopologs are shown as bar graphs. Groups not compared indicate no statistical significance. *n* = 3 technical replicates per group; 1-way ANOVA corrected for multiple comparisons with Tukey’s multiple comparisons test. *P < 0.05; **P < 0.01; ***P < 0.001. Data are mean ± SD.

Fatty acid is another source for both energy metabolism and biosynthetic intermediates for T cell growth and memory differentiation (13, 55). Thus, we traced ^13^C palmitate as a carbon source to understand how PDK1 and PDP1 transduction changed the way cells used and accumulated fatty acid–derived carbons. Similar to the glutamine-tracing experiment, the activated CD8^+^ T cells were incubated with ^13^C palmitate for 1 or 6 hours before the cells were analyzed. First, we observed slightly increased M+14-labeled palmitate in PDK1 and PDP1 groups compared with EV controls, especially at 1 hour (Figure 5A). A similar trend of increased fatty acid accumulation in PDK1 and PDP1 groups was observed in the M+16 palmitate without statistical significance (Supplemental Figure 7A). Tetradecanoic acid and octadecanoic acid, 14- and 18-carbon saturated fatty acids, were also detected with minor changes in the PDP1 group without statistical significance (Supplemental Figure 7, B–E). Interestingly, reduced acylcarnitine accumulation in PDK1 with various lengths of 2, 14, and 16 carbons was observed while PDP1 induced acetylcarnitine enrichment (Figure 5, B–D). These results suggest that the fatty acid–derived carbons were more actively broken down in PDP1-transduced cells.

Once the acylcarnitine is transported into mitochondria, the acyl-CoA is broken down into multiple acetyl-CoA molecules after β-oxidation. The resulting acetyl-CoA can be used for de novo lipid synthesis or energy generation in the TCA cycle (55). Different levels of TCA cycle intermediates were observed in the PDK1 and PDP1 groups. Citrates with different labeling from M+3 to M+5 were decreased in the PDK1 group, while more M+4 citrates were labeled after 6 hours of incubation in PDP1 (Figure 5, E and F). M+2 citrate did not show significant changes but followed the same trend (Supplemental Figure 7F). In the later part of the TCA cycle, M+4-labeled malate levels were also higher at 1 and 6 hours in the PDP1 group than PDK1 group (Figure 5G). Succinate and fumarate followed a similar pattern of decreased palmitate-derived ^13^C labeling with PDK1 and relatively higher labeling in PDP1 transduction without statistical significance (Supplemental Figure 7, G and H). Interestingly, M+5 glutamate levels were higher in the PDP1 group than in the other 2 groups (Figure 5H).

These data indicate enhanced fatty acid metabolism in the PDP1 group, which contributes to the production of TCA cycle intermediates. Increased glutamate labeling may indicate palmitate-derived carbons being diverted to glutamate production, as there is already a sufficient supply of intermediates to fuel the TCA cycle (21).

These ^13^C palmitate-tracing results suggest that fatty acid oxidation and TCA cycle incorporation were differentially affected by the transduction of PDK1 versus PDP1 despite similar overall mitochondrial respiration observed in both groups (Figures 2 and 3).

### PDK1- and PDP1-transduced CD8^+^ T cells show enhanced primary response in mice with MHV-68 infection.

**Figure 6:**
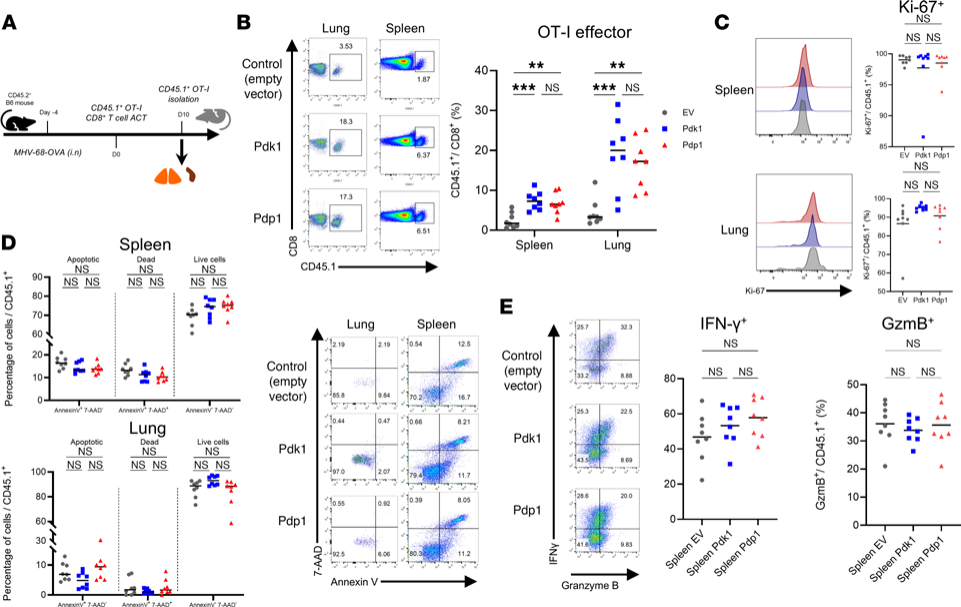
Engineered OT-I CD8^+^ T cells induce an enhanced primary response upon MHV-68 challenge. (**A**) Wild-type CD45.1^+^ OT-I T cells retrovirally transduced by EV, PDK1, or PDP1 before adoptive transfer into CD45.2^+^ C57BL/6 mice infected by MHV-68-OVA 4 days before ACT. (**B**–**D**) At 10 days later, primary effector CD45.1^+^ OT-I cells, proliferation marker Ki-67, and apoptosis/cell death markers annexin V/7-aminoactinomycin D (7-AAD) staining from spleens and lungs were measured by flow cytometry. (**E**) Production of effector molecules IFN-γ and GzmB after SIINFEKL peptide stimulation. Corresponding flow cytometry plots and dot plots are shown. Experiments were repeated 3 times. *n* = 8 biologically independent samples per group; 1-way ANOVA corrected for multiple comparisons with Tukey’s multiple comparisons test. **P < 0.01; ***P < 0.001.

To test the role of enforced PDK1 or PDP1 expression on the T cell response in vivo, we elected to use the MHV-68 model system, a persistent virus infection model that induces a potent CD8^+^ T cell response does not subject T cells to exhaustion (56). We adoptively transferred transduced OT-I CD8^+^ T cells into B6 mice infected by MHV-68 encoding OVA, which was recognized by the OT-I transgenic T cell receptor. Recipient CD45.2^+^ mice were infected with MHV-68-OVA 4 days before the adoptive cell transfer (ACT) of transduced OT-I cells (Figure 6A). Spleens and lungs were harvested during the primary response to measure the cell expansion and phenotype with flow cytometry. We first analyzed the magnitude of the primary splenic CD8^+^ T cell response at day 10 after transfer by measuring the proportion of CD45.1^+^ OT-I cells among live CD8^+^ cells. The CD8^+^ T cell responses were consistently higher in both of the groups compared with EV in spleens and lungs (Figure 6B and Supplemental Figure 8, A and B). To test if these differences were attributable to cell proliferation, we measured the Ki-67 protein as an indicator of cell division. Neither PDK1 nor PDP1 populations showed significant changes in Ki-67 staining (Figure 6C). PDK1 and PDP1 groups in the lung had a marginally increased proportion of Ki-67^+^ cells. Regarding cell survival and apoptosis, annexin V and 7-aminoactinomycin D (7-AAD) staining did not show significant changes in the proportions of early apoptotic or live cell populations (Figure 6D). The differences in T cell population sizes could not be attributed solely to more proliferation or cell survival but were likely due to small changes in both attributes. To evaluate T cell function, we measured the production of effector molecules granzyme B (GzmB) and IFN-γ after 5 hours of stimulation with cognate antigen. Similar proportions of PDK1- and PDP1-transduced T cells produced effector molecules when compared with EV cells, indicating in vivo effector functions were not compromised (Figure 6E). We also conducted experiments to measure MHV-68-OVA virus titers from the lungs of the infected mice by viral plaque assay. As viral titers peak days 5–7, to allow sufficient time for T cell activity to be manifest, T cells were adoptively transferred 2 days after MHV-68 infection in these experiments. The viral loads were not significantly different between the engineered and the control T cell groups (Supplemental Figure 9), indicating all groups mounted T cell responses robust enough to control the infection to an equivalent degree. These results show that in MHV-68 infection, the T cell effector response was larger in magnitude with PDK1 or PDP1 expression, and there was no detrimental impact on effector functions.

### Memory CD8^+^ T cells with PDK1 or PDP1 transduction showed enhanced secondary responses after MHV-68 rechallenge.

We measured the efficiency with which each T cell population established memory by detecting donor cells 30 days after adoptive transfer. In lungs and spleens, all 3 groups of transduced OT-I cells established memory similarly (Supplemental Figure 10A). This is consistent with the similar early differentiation of memory precursor effector cells and short-lived effector cells during the day 10 effector peak (Supplemental Figure 10B).

**Figure 7:**
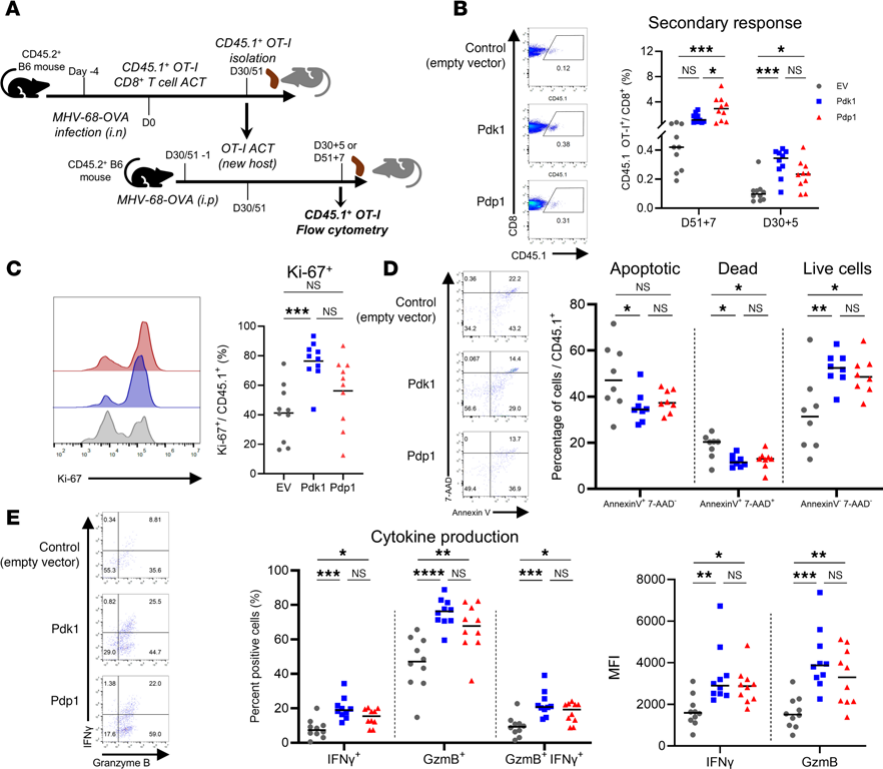
Engineered OT-I memory CD8^+^ T cells induce an enhanced secondary response after MHV-68 challenge. (**A**) Day 30–51 memory CD8^+^ OT-I cells from MHV-68-OVA–infected mice were isolated and transferred into naive recipient B6 mice infected by MHV-68-OVA 1 day before ACT, and the secondary effector cells were characterized after 5 or 7 days by flow cytometry. (**B**–**E**) CD45.1^+^ OT-I cell expansion, expression of Ki-67, apoptosis/cell death marker annexin V/7-AAD, and production of effector molecules IFN-γ and GzmB after SIINFEKL peptide stimulation. Graphs show the proportion of cells positive for effector molecules and geometric mean fluorescence intensity (gMFI). Experiments were repeated 3 times. *n* = 8–10 biologically independent samples per group; 1-way ANOVA corrected for multiple comparisons with Tukey’s multiple comparisons test. *P < 0.05; **P < 0.01; ***P < 0.001; ****P < 0.0001.

Next, we tested whether our metabolic engineering approach affected the quality of the memory CD8^+^ T cell response. To interrogate the function of memory CD8^+^ T cells, we tested the recall response upon secondary antigenic challenge. The transduced OT-I cells were transferred into MHV-68-OVA–infected mice, similar to the primary immune response model. Instead of harvesting the cells during the peak of the effector response, transferred cells were allowed to establish memory for 30–51 days after transfer (Figure 7A). Memory OT-I cells were isolated from the spleen, and the CD45.1^+^ cells were transferred into CD45.2^+^ recipients infected with the same virus 1 day prior. Memory cells were allowed to mount the secondary effector responses for 5 to 7 days after transfer.

Secondary effector responses were measured by flow cytometry by detecting CD45.1^+^ OT-I cells from the spleen. The clonal expansion of CD45.1^+^ cells was larger in the secondary responses in PDK1 and PDP1 groups compared with EV (Figure 7B). Levels of the proliferation marker Ki-67 were also higher, especially in PDK1, indicating greater proliferation drove the larger secondary effector CD8^+^ T cell expansion (Figure 7C). Also, cell survival and apoptosis were measured by flow cytometry staining for annexin V and 7-AAD, markers for apoptosis and cell death, respectively. Among CD45.1^+^ OT-I cells, the proportion of dead cells (annexin V^+^7-AAD^+^) was higher in EV compared with the 2 other groups (Figure 7D). There was also a reduction in early apoptotic cells (annexin V^+^7-AAD^–^) in the PDK1 and PDP1 groups. There was a corresponding increase in live cells (annexin V^–^7-AAD^–^), suggesting that the PDK1 and PDP1 transduction induced enhanced cell survival and reduced apoptosis in CD8^+^ T cells after antigenic rechallenge. Interestingly, the cells in the secondary response showed different levels of effector molecule GzmB and cytokine IFN-γ. The PDK1 and PDP1 group cells showed higher levels of cytokine production and higher proportions of cells producing effector molecules, indicating better functional responses with the engineered memory cells (Figure 7E). Consistent with the enhanced primary immune response, the quality and quantity of the transduced CD8^+^ T cells were improved in the memory recall response.

### CD8^+^ T cells transduced with PDK1 or PDP1 show compromised protection against mouse B cell lymphoma, which is rescued by glutamine and acetate supplementation.

As immunosuppressed patients infected with gammaherpesviruses can develop lymphoma (57, 58), we investigated the role of engineered CD8^+^ T cells in the control of the physiologically relevant EμMyc B cell lymphoma. Our previous studies have shown that MHV-68–induced memory cells protect mice against the growth of this tumor when tumor cells express a native viral antigen (28). In the current study, the EμMyc B cell lymphoma cell line was retrovirally transduced with OVA antigen and luciferase to be targeted by OT-I cells and to allow tumor monitoring following luciferin injection in vivo.

**Figure 8:**
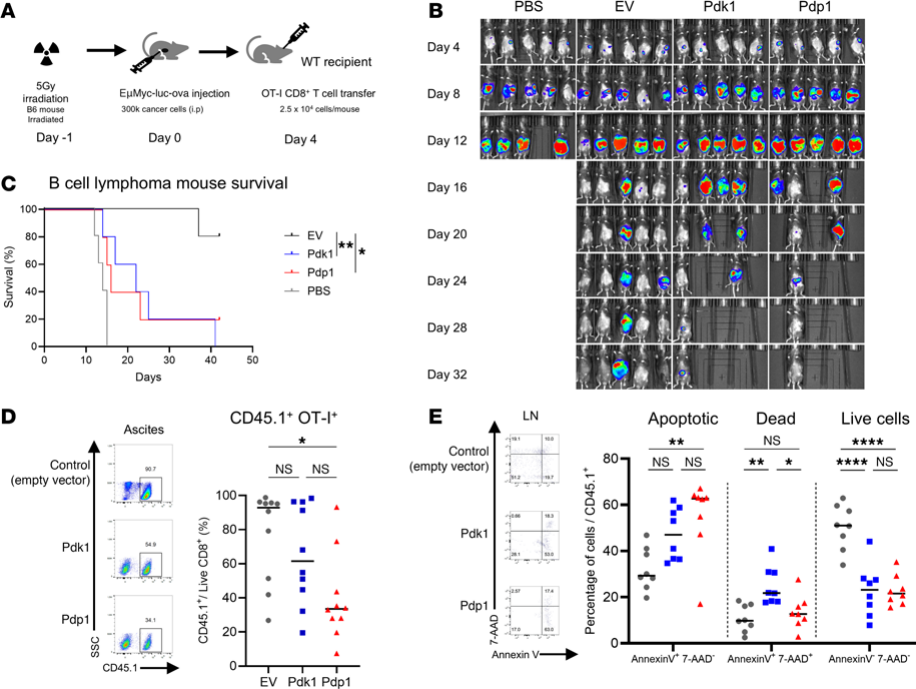
CD8^+^ T cells overexpressing PDH regulatory enzymes show compromised efficacy against tumor challenge. (**A**) B6 mice received 300,000 EμMyc-OVA B cell lymphoma cells expressing luciferase by intraperitoneal injection. Then, 4 days later, 25,000 transduced CD8^+^ T cells (EV/Pdk1/Pdp1) were adoptively transferred into B lymphoma–bearing mice. (**B**) In vivo IVIS imaging of mouse tumors over the course of 32 days. (**C**) Kaplan-Meier survival analysis of the tumor-bearing mice showing significant reductions in PDK1 (*P* = 0.0064 Mantel-Cox log-rank test) and PDP1 (*P* = 0.0316) groups compared with EV. (**D**) Flow cytometry analysis of OT-I cells in vivo. A total of 1 million EμMyc-OVA tumor cells were injected into B6 mice, followed by 500,000 transduced OT-I CD8^+^ T cells 4 days later. T cells were analyzed by flow cytometry 6 days after adoptive transfer. (**E**) Apoptosis and cell death marker staining of OT-I cells from inguinal lymph nodes of the B cell lymphoma model. Experiments were repeated 2 times. *n* = 5–10 biologically independent samples per group; 1-way ANOVA corrected for multiple comparisons with Tukey’s multiple comparisons test. *P < 0.05; **P < 0.01; ****P < 0.0001. Data are mean ± SD.

B6 mice were irradiated with a sublethal dose of radiation (5 Gy) 1 day before tumor injection to induce lymphodepletion, mimic the immunocompromised condition, and better define the efficacy of transferred antigen-specific CD8^+^ T cells. After 1 day, EμMyc B cell lymphoma cells were injected intraperitoneally into the mice. After 4 days, when tumors started growing in the peritoneum, transduced OT-I CD8^+^ T cells or PBS were adoptively transferred into tumor-bearing mice (Figure 8A).

Mouse survival and tumor growth in the peritoneum were monitored every 4 days in vivo by luciferin injection (Figure 8, B and C). In the initial 8 to 12 days, tumors grew aggressively in all experimental groups. After this phase, OT-I T cell–mediated immunity controlled the tumors while mice without OT-I T cells (PBS) all died (Figure 8B). While most of the mice in the PDK1 and PDP1 groups either died or had easily detectable tumors by day 16, mice in the EV group controlled the tumor better. Survival was also worse in the recipients of OT-I cells transduced with PDK1 or PDP1 compared with EV (Figure 8C).

Next, we analyzed the OT-I cells from the ascites of the tumor-injected mice. There was no difference in the frequencies of exhausted OT-I cells in the ascites (Supplemental Figure 11A). Higher frequencies of apoptotic and dead cells were detected in draining lymph nodes in the PDK1 and PDP1 groups, relative to the control (Figure 8E), but not in the ascites (Supplemental Figure 11B). The proportion of proliferating cells and cells capable of producing IFN-γ or GzmB was not significantly different in either the ascites or lymph nodes (Supplemental Figure 11, C–E). These data indicate PDK1 and PDP1 retained effector and proliferative activity but were deficient in survival in draining lymph nodes.

Therefore, while PDK1- and PDP1-transduced CD8^+^ T cells mounted better responses in the virus infection model (Figures 6 and 7), their antitumor responses were compromised (Figure 8). This may be due to the restricted metabolic flexibility of CD8^+^ T cells by the enforced expression of genes regulating PDH activity, and this is more critical in a tumor than in a virus infection model.

**Figure 9:**
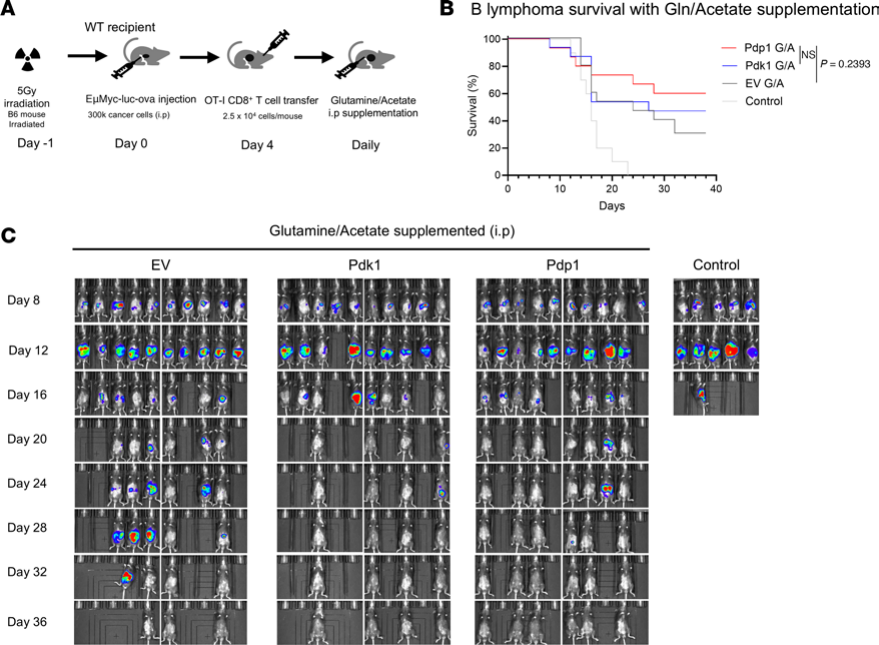
Glutamine and fatty acid supplementation improves tumor protection with engineered OT-I T cells. Using the same EμMyc-OVA mice as described in Figure 8, (**A**) glutamine (200 mg/kg) and sodium acetate (1 g/kg) were intraperitoneally delivered into tumor-bearing mice for glutamine and fatty acid supplementation. Supplementation was given daily from day 4 to the endpoints. (**B**) Kaplan-Meier survival analysis of the tumor-bearing mice with nutrient supplementation showed enhancement of T cell protective capacity in PDK1 (*P* = 0.5964 Mantel-Cox log-rank test) and PDP1 (*P* = 0.2393) groups compared with EV. (**C**) In vivo IVIS imaging of mouse tumors over the course of 36 days. Results were pooled from 2 independent experiments. *N* = 10 (no OT-I control) or *N* = 15 mice per group.

Supplementation with glutamine or the short-chain fatty acid acetate can enhance antitumor immunity (59–62). Our metabolomics data (Figures 4 and 5) indicated PDK1 or PDP1 groups preferentially metabolized glutamine and fatty acids, so we hypothesized that deficiency in these nutrients in the TME may be responsible for the poorer protection observed using these transduced T cells. To test this, we administered daily glutamine and sodium acetate supplements intraperitoneally to EμMyc B cell lymphoma–bearing mice that received CD8^+^ T cells transduced with EV, PDK1, or PDP1 (Figure 9A).

Tumors grew well in all groups receiving glutamine/acetate supplementation for the first 12 days. Interestingly, we observed that the overall survival of the PDK1 and PDP1 groups after glutamine/acetate supplementation was marginally higher than EV control (Figure 9B). We also observed that the mice that survived initial tumor growth after 16 days controlled the tumors better with PDK1- and PDP1-transduced OT-I cells compared with EV until day 30 (Figure 9C). These data indicate that when PDK1- or PDP1-transduced T cells receive plentiful glutamine and acetate, this restores their tumor-protective ability to be at least comparable to and possibly a little better than control CD8^+^ T cells.

## Discussion

Although previous studies have examined the role of PDK1 and glycolysis in CD8^+^ T cell differentiation (23), the consequences of altering the expression of PDK1 and the opposing phosphatase PDP1 in vivo remain to be investigated. The focus of this study was the intentional activation or inhibition of PDH activity to measure effects on effector and memory CD8^+^ T cell responses. Contrary to expectations, we found either enforced PDK1 or PDP1 expression resulted in enhanced CD8^+^ T cell metabolic capacity and increased primary and secondary responses in a virus infection model. Critically, these enhanced responses did not extend to conferring better protection in a B cell lymphoma model, which we ascribe to the absence of key metabolic fuels, illustrating a key role for immunological context in CD8^+^ T cell metabolic reprogramming.

Both the retrovirally transduced *Pdk1* and *Pdp1* genes increased glycolytic and OXPHOS capacity, but they showed different fuel utilization with glutamine and palmitate in CD8^+^ T cells. These engineered cells showed similar phenotypes not only with respect to in vitro metabolism but also after in vivo antigenic challenge. Cells transduced with PDK1 and PDP1 expanded more in both primary and secondary challenges by MHV-68, and this was due to reduced apoptosis and increased proliferation in the secondary response. Although the recall responses to MHV-68 induced more effector molecule production (GzmB^+^IFN-γ^+^) in the engineered cells, the EμMyc B cell lymphoma challenge showed worse tumor protection with larger tumors and shorter survival time in recipients of the engineered cells. This reduced T cell protection was rescued when glutamine and acetate were supplemented to tumor-bearing mice.

Our results demonstrated that enforced expression of PDK1 and PDP1 induced a transcriptional program distinct from EV-transduced cells, while PDK1 and PDP1 groups were transcriptionally similar to each other. Genes induced by the engineering were mostly related to mitochondrial metabolism, cell survival, and proliferation rather than genes relating to other immunological pathways including broader differentiation processes in CD8^+^ T cells. It will be informative to study the transcriptional profile and their heterogeneity in in vivo models, such as tumor and viral infections, in future research.

Two previous studies found that glutamine was redirected into the TCA cycle when pyruvate supply to the mitochondria was reduced (63, 64). Our data indicate that a similar process is occurring in CD8^+^ T cells after PDK1 expression, as increased glutamine-derived TCA cycle intermediates were observed in PDK1-expressing T cells. This previous work supports our observations that CD8^+^ T cells with PDK1 or PDP1 metabolized nutrients differently by utilizing more glutamine or fatty acids, respectively, in the TCA cycle (64). It is noteworthy that different lengths (^13^C_14-18_) of fatty acids, even longer than palmitate, were synthesized marginally higher in PDP1-transduced cells while some of them were lower in the PDK1 group without reaching statistical significance. Combined with the enriched ^13^C_14_ and ^13^C_16_ acylcarnitines and ^13^C_2_ acetylcarnitines in the PDP1 group, the data suggest higher levels of fatty acid oxidation and de novo synthesis are occurring at the same time in the PDP1 group. Meanwhile, the PDP1 group also showed enriched ^13^C citrate and ^13^C glutamate compared with the PDK1 group. This may indicate sufficient intermediates are produced to sustain the TCA cycle in the PDP1 group, so excess carbons can be exported from the mitochondria as citrate or glutamate for use in other metabolic pathways (21).

In addition, citrate from ^13^C lactate was also enriched in the PDP1 group compared with the PDK1 group. It is increasingly appreciated that citrate in immune cells can lead to epigenetic modification via acetyl-CoA (48, 65). In Th1 cells, H3K9 acetylation controls the expression of IFN-γ independent of the 3′UTR (21), which may explain the enhanced IFN-γ responses we observed in the secondary responses to MHV-68. Thus, the consistent differences in labeled citrate levels derived from palmitate, glutamine, and lactate imply that PDP1-transduced cells may have higher histone acetylation activity. PDH activity in the nucleus is another possible regulatory mechanism known to modify H3K27, H3K9, and H3K18 acetylation, increasing expression of cell cycle and activation genes (31, 32). We could detect PDP1 in nuclei as well as mitochondria, consistent with a recent study where PDP1 induced *Hif1α*/*Pdk1* in nuclei under hypoxia (30). This homeostatic loop can control histone acetylation and potentially preserve acetyl-CoA levels in the cell. This suggests an interesting PDP1 regulatory feedback mechanism possible in CD8^+^ T cells, as PDP1 inducing PDK1 and glycolytic metabolism may partly explain the similar phenotypes of PDK1 and PDP1 group CD8^+^ T cells, even though they function in opposite directions and utilize nutrients differently. For maintaining acetyl-CoA levels, other sources, such as acetate, can generate acetyl-CoA and support histone acetylation in effector T cells by acetyl-CoA synthetase under glucose-restricted conditions (66). It is possible that PDK1 and PDP1 groups used glutamine differently for anaplerotic replenishment while citrate from other fuels was used outside of the TCA cycle (67). Since we observed different levels of citrate derived from glutamine, palmitate, and lactate with PDK1 and PDP1 transduction, it will be interesting to investigate how different nutrient sources supply acetyl-CoA and histone acetylation in T cells.

An unanticipated metabolomic result was that ^13^C glucose tracing showed no significant differences in glycolytic and TCA cycle metabolite levels between groups. The presence of palmitate and lactate in media used for metabolomics assays but not Seahorse assays could be a key reason. The availability of lactate and palmitate and their usage by PDK1- and PDP1-transduced cells likely reduced the requirements for glucose, such that glucose utilization was similar to control T cells in metabolomics studies. Meanwhile, the media used in Seahorse assays without lactate and fatty acid showed enhanced glycolysis that was inhibited by 2-DG in both groups.

Enforced expression of PDP1 resulted in increased ECAR readings in Seahorse assays, suggesting some of the increased carbon flux to the mitochondrial TCA cycle ultimately returned to the cytosol. Our data show that ME1 and PEPCK both contribute to this process, converting the TCA cycle intermediate malate to pyruvate. This is consistent with the known role of PEPCK in tumor control and IFN-γ production as the metabolite PEP suppresses SERCA-mediated calcium uptake and sustains cytosolic Ca^2+^/nuclear factor of activated T cells signaling (68). Our result verifies that supporting glycolytic demand from TCA intermediates through PEPCK is important in effector CD8^+^ T cells. Furthermore, PEPCK is known to drive gluconeogenesis and glycogen storage in memory CD8^+^ T cells, generating glucose-6-phosphate in pentose phosphate pathway for NADPH and subsequent GSH production (41). The contribution of PEPCK to the first step of gluconeogenesis in PDP1-transduced CD8^+^ T cells in which TCA cycle flux was increased can further support this concept. Another connection between the TCA cycle and pyruvate regeneration is through malic enzymes (MEs). ME1 is known to increase lactate production and glucose uptake in breast cancer cells, leading to glycolytic metabolism (38). Also, ME2 increases glycolytic flux and lactate production, contributing to osteoblast proliferation and differentiation, for which glucose consumption is important, similar to T cell activation (69). These studies are consistent with our results showing the diversion of TCA intermediates to pyruvate and lactate in PDP1-transduced cells.

A previous study overexpressed the glycolytic gene phosphoglycerate mutase 1 (*Pgam1*), which augmented glycolytic flux, induced terminally differentiated CD8^+^ T cells, and impaired long-term memory in the Vaccinia virus model (11). An important distinction between this report and the current study is where the intervention is in the glycolysis pathway. PGAM1 regulates upstream glycolysis while PDK1 promotes lactate production at the last step of glycolysis. This suggests the steps in between may contribute to the differentiation of T cells. PEP, for instance, is downstream of PGAM1 and enhances effector T cell differentiation. By promoting lactate fermentation and PEP consumption, the accumulation of this metabolite may be alleviated in PDK1 compared with PGAM1-overexpressing cells. The intact memory cells observed with PDK1 overexpression show that interventions in glycolytic metabolism can show diverse memory differentiation phenotypes and outcomes for T cell therapy.

These metabolic phenotypes in PDK1 and PDP1 groups suggest how disruption of PDH homeostasis could converge on similar phenotypes. This unexpected result could be explained by 3 possible mechanisms: (a) Nuclear localization of PDP1 and active PDH can induce PDK1, leading to enhanced glycolytic metabolism to maintain PDH homeostasis. (b) Compensatory pathways connecting mitochondria and glycolysis, such as ME1 and PEPCK, can generate glycolytic metabolites, pyruvate and PEP, to drive converging metabolic phenotypes. (c) Different fuel source utilization, including fatty acid and glutamine, could compensate for changes in PDH activity to meet the metabolic demand, such as TCA anaplerosis by glutamine in the PDK1 group, and may lead to similar phenotypes with respect to enhanced rates of glycolysis and OXPHOS. Enhanced glycolysis and OXPHOS, together with improved effector molecule production, are similar to those seen in secondary effector cells, which likely explains the superior secondary responses we observed in the MHV-68 model (70–72).

Interestingly, the engineered CD8^+^ T cells in the B cell lymphoma model showed worse protective efficacy as opposed to enhanced T cell responses in MHV-68 infection. These contrasting results emphasize the importance of the nutrient microenvironment in the metabolic reprogramming of T cells. A study focusing on inhibition of mitochondrial pyruvate carrier (MPC) detailed the seemingly contradictory role of pyruvate metabolism via the TCA cycle (64). MPC inhibition imprinted a pro-memory phenotype through acetyl-CoA synthesis from glutamine and fatty acids, but MPC was essential for lactate oxidation, supporting CD8^+^ T cell antitumor function. Therefore, pyruvate metabolism plays a multifaceted role in the TME, which may explain why restrictions on pyruvate usage may be detrimental. In the MPC study, preconditioning with an MPC inhibitor before in vivo administration resulted in effective and persistent CAR-T cells. Glutamine and acetate supplementation can inhibit tumor growth by affecting T cell immunity, conventional type 1 dendritic cells, metabolism within TME, and epigenetics (59–62). Here, we show this supplementation can restore compromised antitumor functions in T cells with targeted metabolic enhancements. Thus, the enforced expression of PDH phosphorylation regulators in the current study could have constrained the metabolic flexibility in CD8^+^ T cells, illuminating the importance of metabolic adaptation in the TME.

Under hypoxia, PDK1 is required for metabolic adaptation by attenuating mitochondrial ROS in the hypoxic environment (73). In this context, PDK1 is necessary for preventing electron leakage from the inefficient TCA cycle and permits continued glycolysis for cell survival. Pyruvate carboxylase (PC) is also important in anaplerosis for succinate secretion and T cell effector function while PDH inhibition improved cytotoxicity by increasing PC activity (33). These findings suggest that with a limited nutrient supply, glycolysis and PC activity facilitating anaplerosis can be important in fine-tuning the metabolic balance in the TME. Research on mitochondrial phosphatase PTPMT1-knockout CD8^+^ T cells discovered enhanced fatty acid and glutamine utilization while glucose and pyruvate utilization were inhibited. Persistent mitochondrial inflexibility in this fuel selection induced oxidative stress, exhaustion, and impaired antitumor immunity in CD8^+^ T cells (74). From previous studies and our findings, metabolic plasticity and the ability to adapt to fuel availability are essential for T cell tumor therapy (75). For future therapeutic approaches, ex vivo conditioning of CD8^+^ T cells for metabolic manipulation may be an alternative approach that better preserves metabolic flexibility (11, 64). It is noteworthy that a recent study by Frisch et al. took a pharmacological approach with dichloroacetate (DCA) to temporarily inhibit PDK1 in vitro, which improved T cell efficacy against cancer accompanied by increased stemness characteristics and survival of T cells (76). Consistent with our result, prolonged intervention with DCA treatment in mice negated the therapeutic efficacy, which highlights the finding that long-term enforcement of pyruvate conversion pathways may not be the best immunotherapeutic approach for tumors.

The current study also focused on T cell metabolism during infection with gammaherpesvirus MHV-68 as a model. With increasing evidence accumulating in the past 2 years, the correlation between the human gammaherpesvirus Epstein-Barr virus and multiple sclerosis shows the importance of translational approaches for immune responses to persistent viruses (77–79). Alteration of T cell metabolism has been shown in several types of virus infection. SARS-CoV-2 reduces glycolytic activity and glucose transporter 1 expression and causes mitochondrial dysfunction in T cells (16). Chronic viruses, such as HIV, can also affect T cells by inducing exhaustion and altering metabolism by promoting glycolysis, which permits HIV replication in CD4^+^ T cells (15). T cell–mediated immunity against influenza infection depends on CD4^+^ T cells to provide help shape CD8^+^ T cell metabolism (27). Other immune cells are also metabolically affected during infections, including highly glycolytic myeloid-derived suppressor cells that inhibit CD4^+^ T cell glycolytic activity by lactate secretion in *Staphylococcus aureus* infection (80). Our study and future research on the metabolism of immune cells beyond CD8^+^ T cells will be important in understanding how infection and the local environment affect the immune landscape.

Although this study demonstrated changes in murine CD8^+^ T cell response against different types of antigenic challenges, we did not study human immune responses. Future studies on human T cells against various infections and cancers will provide a fuller understanding of the role of pyruvate metabolism. Our study was also limited in that only retroviral transduction was used to manipulate metabolic pathways. Other pharmacological and genetic approaches will help provide more insights and move immunometabolic intervention closer to the clinic. As other isoforms of PDK and PDP are less studied in T cells (81–85), studying the combinatorial effect of different isoforms, including splicing variants of *Pdp1* as well as the balance of PDK versus PDP, will be interesting. Although our study has discussed interesting immunological phenotypic changes, a deeper understanding of the mechanisms of transcriptional programming and epigenetic remodeling will be important.

An important implication of this study is that therapeutic strategies utilizing metabolic intervention should consider the essential role of the nutrient microenvironment where T cells are required to exert effector function. We predict that allowing metabolic flexibility would enhance the therapeutic potential of T cells, allowing them to adapt to the metabolic environment encountered in the relevant disease.

Finally, we found that enforced expression of PDH phosphoregulatory genes in CD8^+^ T cells enhanced metabolic capacity and T cell responses against viral infection, but they led to a less protective antitumor response. This study will provide insights into the PDH complex as a key step of T cell metabolism for controlling the balance of glycolysis and OXPHOS, suggesting the importance of nutrient environment adaptation and the necessity of fine-tuning T cell metabolism for therapeutic purposes.

## Methods

### Sex as a biological variable.

We conducted this study on MHV-68 infection and B cell lymphoma with both male and female mice.

### Mice.

Female C57BL/6NCrl mice were purchased from Charles River Laboratories. CD45.1 (Jackson Laboratory, 002014) and OT-I (Jackson Laboratory, 003831) female mice with wild-type C57BL/6 backgrounds were originally purchased from Jackson Laboratory. These 2 mouse strains were crossed, and CD45.1 OT-I mice were maintained in-house at Dartmouth College. Mice of 8–12 weeks of age were used for experiments, and OT-I mice were backcrossed to B6 mice every other generation.

### Virus.

MHV-68 virus encoding OVA (MHV-68-OVA) was provided by P. Stevenson (University of Queensland, Brisbane, Queensland, Australia). For the primary response, 4,000 PFU of MHV-68 was resuspended in 30 μL of PBS before being intranasally instilled. For the secondary response, 1 million PFU of MHV-68 was resuspended in 100 μL of PBS before intraperitoneally injected.

### Transfection and retrovirus harvest.

Three million HEK293T cells (from Patricia Ernst, University of Colorado Anschutz Medical Campus) were plated on a 10 cm tissue culture dish 24 hours before transfection. A total of 3 μg of retroviral vector and the same amount of packaging vector pCL-Eco DNA plasmids were used for transfection. HEK293T cells were transfected using a calcium phosphate transfection protocol. Medium containing retrovirus was harvested 48 hours after transfection. Transfection efficiency was measured by staining with anti-mouse CD19 antibody (BioLegend, product 115512, clone 6D5). The harvested virus was concentrated using Retro-X concentrator (Takara, 631456), according to the manufacturer’s protocol. The concentrated retrovirus was resuspended in T cell culture medium before being frozen.

### Mouse CD8^+^ T cell culture and retroviral transduction.

Mouse CD8^+^ T cells were isolated from splenocytes using EasySep Mouse CD8^+^ T cell isolation kits (STEMCELL Technologies, 19853). Isolated CD8^+^ T cells were cultured for 48 hours at 500,000 cells/mL/well with T cell culture media (86) with 5 μg/mL of anti-CD28 antibody (BioXcell, BE0015-1) and 25 U/mL recombinant human IL-2 (NIH National Cancer Institute [NCI]; TECIN) on 24-well plates precoated with 10 μg/mL of InVivoMAb anti-mouse CD3 (BioXcell, BE0002) antibody overnight at 4°C.

Activated cells were washed and transduced as previously described (87, 88). Briefly, 3 million cells were mixed with 1 mL concentrated retrovirus, 2 μg/mL Lipofectamine 2000 (Thermo Fisher Scientific), and 1.6 μg/mL polybrene. Cultures were centrifuged at 235*g* at 32°C for 90 minutes. Cells were incubated at 37°C with 25 U/mL recombinant human IL-2 (rhIL-2). At 16 hours after transduction, the cells were stained with anti-mouse CD19 antibody, and the transduced cells were positively enriched with MojoSort Mouse anti-APC Nanobeads (BioLegend, 480072). Transduction efficiency and CD19^+^ cell purity were checked using a Beckman Coulter Cytoflex S cytometer. The cells were then cultured with T cell culture medium and were counted daily to adjust the cell concentration to 0.5–1.0 million cells/mL until they were used for experiments.

### Adoptive transfers and infection.

Mice were infected with MHV-68-OVA 4 days before CD8^+^ T cell adoptive transfer for the primary response and 1 day before transfer for the secondary response.

Cells were cultured with 25 U/mL of rhIL-2, counted, and collected before adoptive transfer. A total of 20,000 cells in 100 μL were retro-orbitally injected into B6 MHV-68-OVA–infected mice.

### Metabolic analysis.

Seahorse assays were performed with an XFe96 Analyzer according to the manufacturer’s protocols (Agilent Technologies). XF cell culture microplate wells were coated with 20 μL of 50 μg/mL poly-d-lysine (Gibco, A38904-01) per well for 1 hour. Poly-d-lysine was washed and dried before use. Seahorse XF base medium without phenol red (Agilent 103575-100) supplemented with 2 mM glutamine, 10 mM glucose, 1.2 mM pyruvate, and 5 mM HEPES was used as assay media. A total of 150,000 cells were seeded per well in the XF cell culture Microplates. We used 1 μM oligomycin, 1 μM FCCP, and 0.5 μM rotenone/antimycin A for the mitostress test and glycolytic rate assay. cPEPCK inhibitor (Axon Mechem, 1165) at the 25 μM concentration (40) and ME1 inhibitor (MedChemExpress, HY-124861) at the 50 μM concentration (89) were used for each Seahorse assay involving pathway inhibition.

### Tissue preparation.

Single-cell suspensions were prepared from spleens, lungs, and inguinal lymph nodes by macerating tissues through nylon filters (Bally Ribbon Mills). Peritoneal cells in the ascites were collected by lavage of the peritoneal cavity with 10 mL of PBS. Red blood cells in the samples were lysed, and the samples were washed and resuspended in PBS containing 2% FBS v/v and 1 mM EDTA before being stained for markers.

### Generation of B cell lymphoma line, in vivo challenge, and imaging.

EμMyc B cell lymphoma cells on a C57BL/6 background (28) were transduced with cloned pCIGAR retroviral vector (from Yina Huang at Dartmouth College) encoding luciferase-mCherry and OVA-eGFP for luciferase and OVA expression. The cells were transduced using 5 μg/mL polybrene and cultured in T cell culture medium before mCherry^+^eGFP^+^ cells were purified using an SH800S Cell Sorter (Sony Biotechnology).

Before tumor challenge experiments, recipient mice were irradiated with 5 Gy radiation. Transduced EμMyc-Luc-OVA cells were injected into C57BL/6 recipient mice intraperitoneally. For mouse survival and tumor growth experiments, 300,000 cells were injected per mouse, and 25,000 transduced OT-I cells were retro-orbitally injected 4 days later. For in vivo tumor imaging, mice were administered 3 mg of d-luciferin (GoldBio, LUCNA) intraperitoneally followed by a 2-minute period before acquiring luminescence image using a Xenogen IVIS-200 imaging system (PerkinElmer). For ascites T cell analysis, 1 million EμMyc-Luc-OVA cells were injected after irradiation and 500,000 transduced OT-I cells were retro-orbitally injected 4 days later. Ascites were harvested 6 days after OT-I adoptive transfer by rinsing the peritoneum of euthanized mice with 10 mL PBS.

Sodium acetate (VWR, 0602-500G) and l-glutamine (Corning, 25-005-CI) were diluted in PBS for 200 mg/kg glutamine and 1 g/kg acetate in vivo supplementation experiment. The same EμMyc-OVA model was used with daily intraperitoneal injection of glutamine and acetate from OT-I adoptive transfer until the endpoint.

### Statistics.

Statistical analyses and graph visualization were performed using GraphPad Prism 10. Statistical tests and multiple comparisons used are described in the figure legends and include 1-way ANOVA with Tukey’s multiple-comparison test and Kaplan-Meier survival analysis with Mantel-Cox long-rank test. A *P* value less than 0.05 was considered significant. Data represent mean ± SD.

### Study approval.

All animal experiments were approved by the Institutional Animal Care and Use Committee of Dartmouth College.

### Data availability.

RNA-Seq data will be made available under GEO accession GSE279041.

## Author contributions

TK, YKU, and EJU conceptualized the project. TK, YKU, SCK, AFV, FWK, AD, HNH, and EJU designed experiments. TK, YKU, JRR, SCK, and RCH performed experiments. TK, JRR, SCK, RCH, OMW, AMH, and EJU analyzed the data. TK, SCM, PCR, AD, HNH, and EJU discussed data interpretation. TK, JRR, OMW, and EJU prepared the manuscript.

## Supplementary Material

Supplemental data

Supplemental data set 1

Supplemental data set 2

Unedited blot and gel images

Supporting data values

## Version 1 08/22/2025

Electronic publication

## Version 2 08/29/2025

Supplemental methods added to supplemental data file
