# Supplementary material for: Targeting pyruvate metabolism generates distinct CD8+ T cell responses to gammaherpesvirus and B lymphoma
Source: JCI Insight. 2025 Aug 22;10(16):e187680. doi: 10.1172/jci.insight.187680 (PMC12406717; doi:10.1172/jci.insight.187680)
Supplement: Supplemental data [file jciinsight-10-187680-s294.pdf]

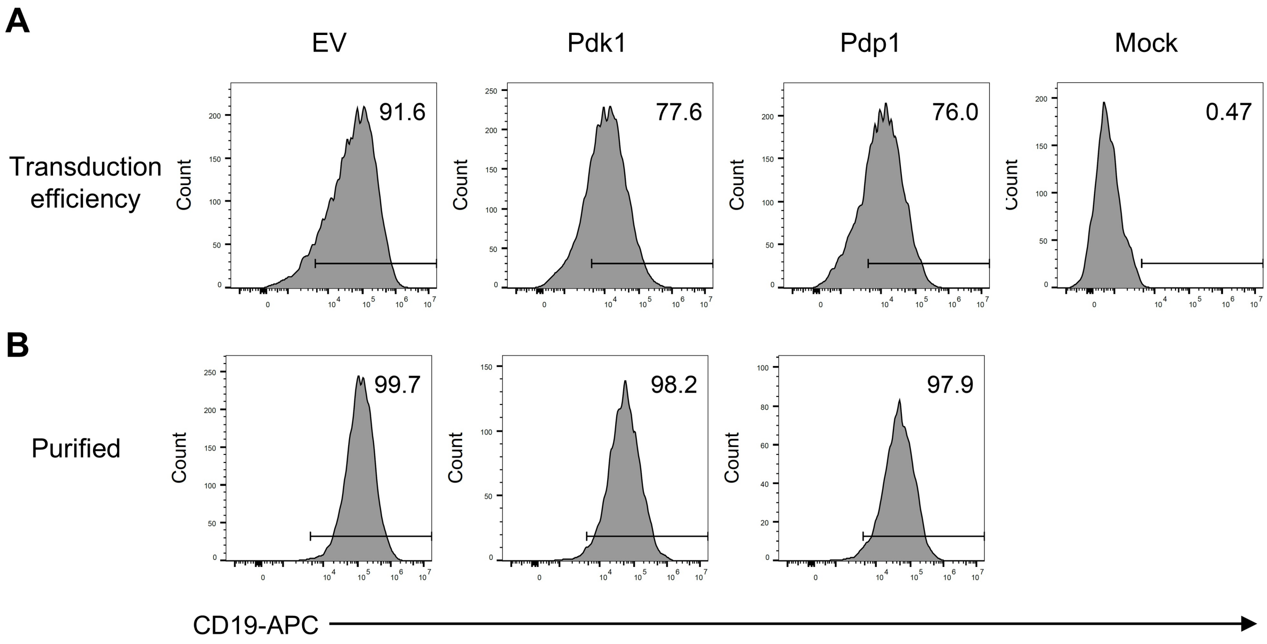

Supplemental Figure 1. CD8⁺ T cell retroviral transduction efficiency and purity CD8⁺ T cells were transduced by retroviruses encoding *Pdk1*, *Pdp1*, or the empty vector (EV). The retroviral vectors expressed truncated mouse CD19 as a reporter. Before being used for experiments, the transduced cells were magnetically purified using anti-CD19 APC antibodies. **(A)** Before the cells were magnetically enriched, the transduction efficiency of CD8⁺ T cells was measured by staining for CD19 expression. **(B)** CD19-APC⁺ purified cells showing over 97% purity.

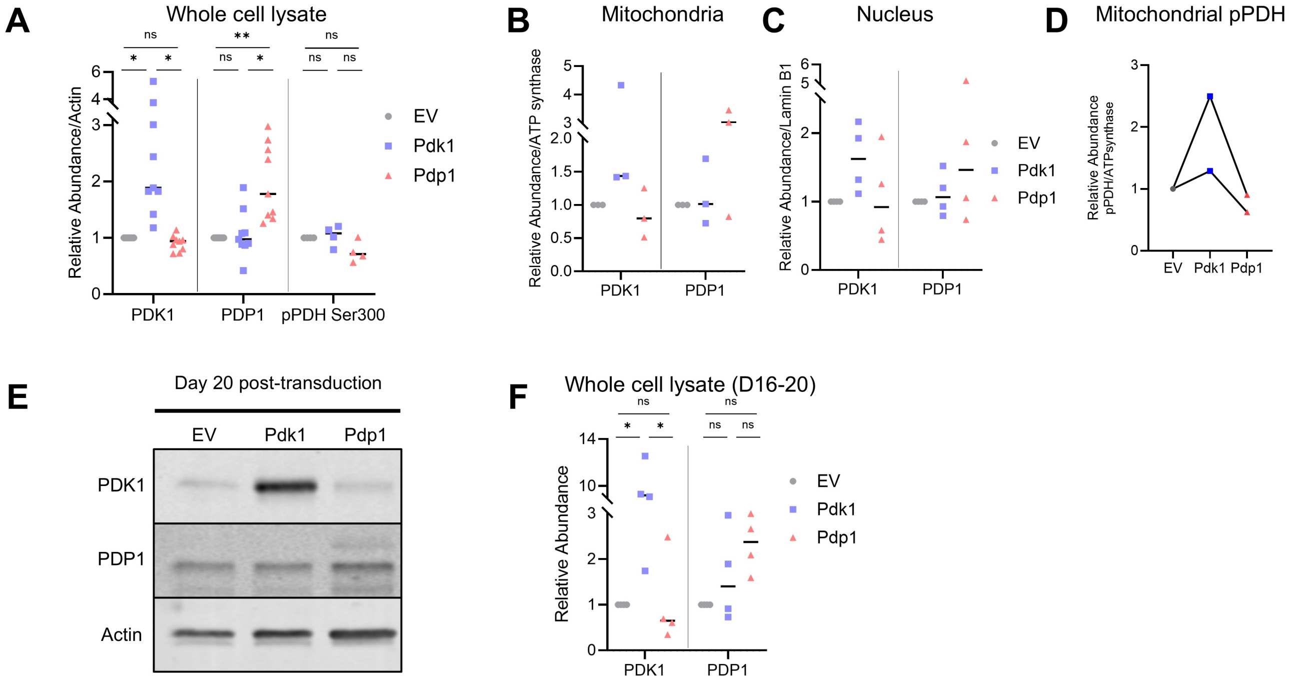

Supplemental Figure 2. PDK1 and PDP1 expression in transduced CD8⁺ T cells CD8⁺ T cells were transduced by retroviruses encoding *Pdk1*, *Pdp1*, or the empty vector (EV), and protein expression was detected as described in Figure 1. Transduced protein overexpression was confirmed from **(A)** whole cell lysate, **(B-C)** mitochondrial and nuclear fractions, shown as graphs where each dot represents an independent western blot result. **(D)** Altered PDH Ser300 phosphorylation with *Pdk1* or *Pdp1* was measured from the mitochondrial fraction. **(E-F)** Sustained expression of the transduced protein expression confirmed from whole lysates of the transduced CD8+ T cells cultured in vitro for 20 (n=3) or 16 (n=1) days after transduction, shown in **F** as graphs where each dot represents an independent western blot result. Experiments were repeated 2-9 times per group with biologically independent samples; One-way ANOVA corrected for multiple comparisons with Tukey's multiple comparisons test. *p<0.05; **p<0.01; ns, not significant.

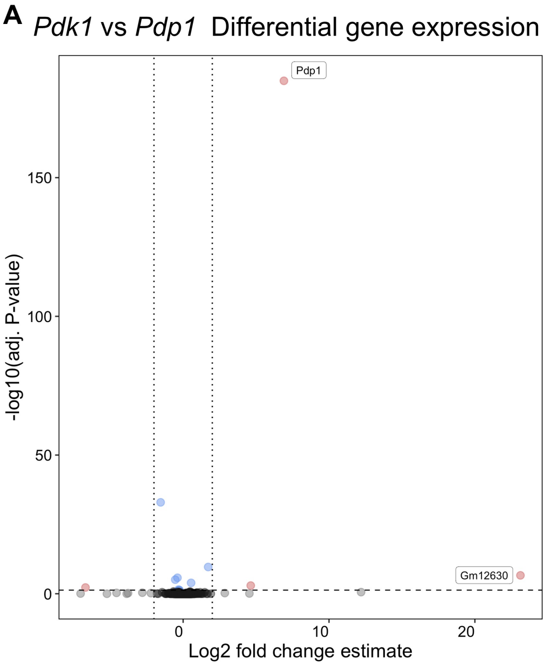

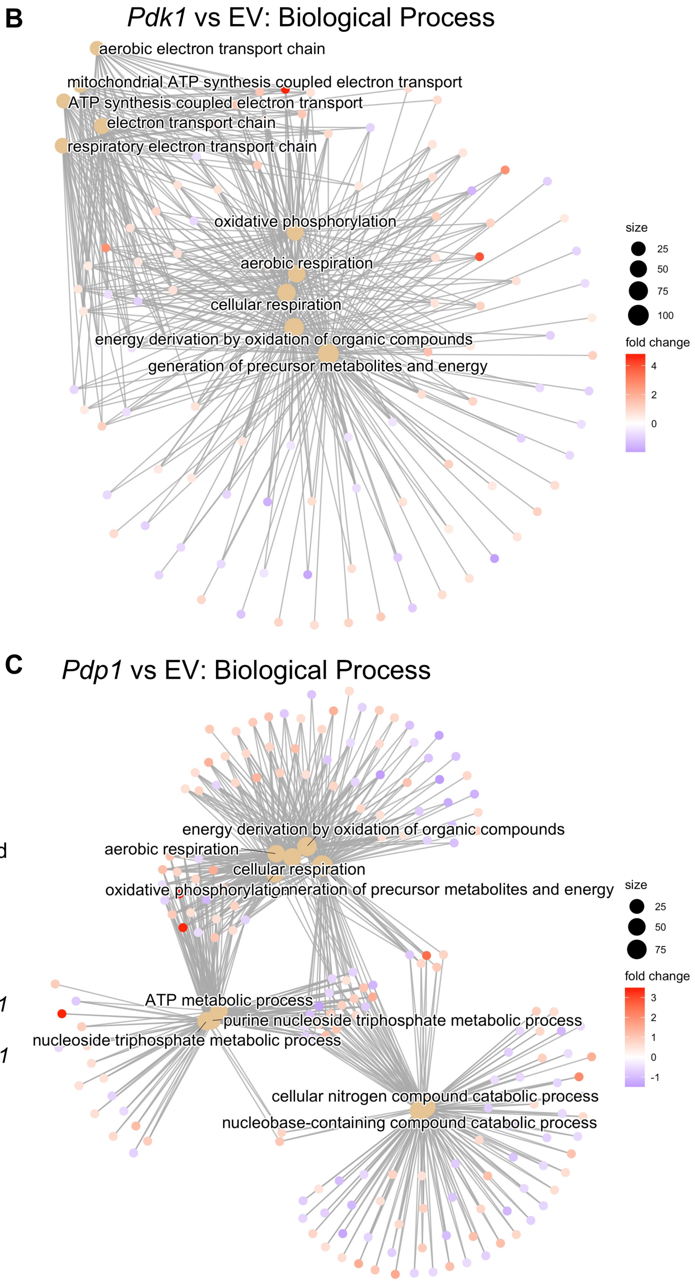

Supplemental Figure 3. RNA-seq transcriptional profile shows similar changes in *Pdk1*- and *Pdp1*-transduced CD8⁺ T cells CD8⁺ T cells transduced by retroviruses packaging *Pdk1*, *Pdp1*, or the empty vector (EV) were cultured in vitro with IL-2 and harvested after 4 days. **(A)** Volcano plots showing DEGs between *Pdk1* and *Pdp1* groups. **(B-C)** Visualization of categories of metabolism-related enriched pathways in the *Pdk1* and *Pdp1* groups by gene-concept networks. **(B)** Top 10 out of 30 enriched pathways from Biological Process gene sets compared between EV and *Pdk1* groups. **(C)** Top 10 out of 20 enriched pathways from Biological Process gene sets compared between EV and *Pdp1* groups. Extending from each pathway node are additional nodes, representing the genes that exist in both the pathway and our differentially expressed genes.

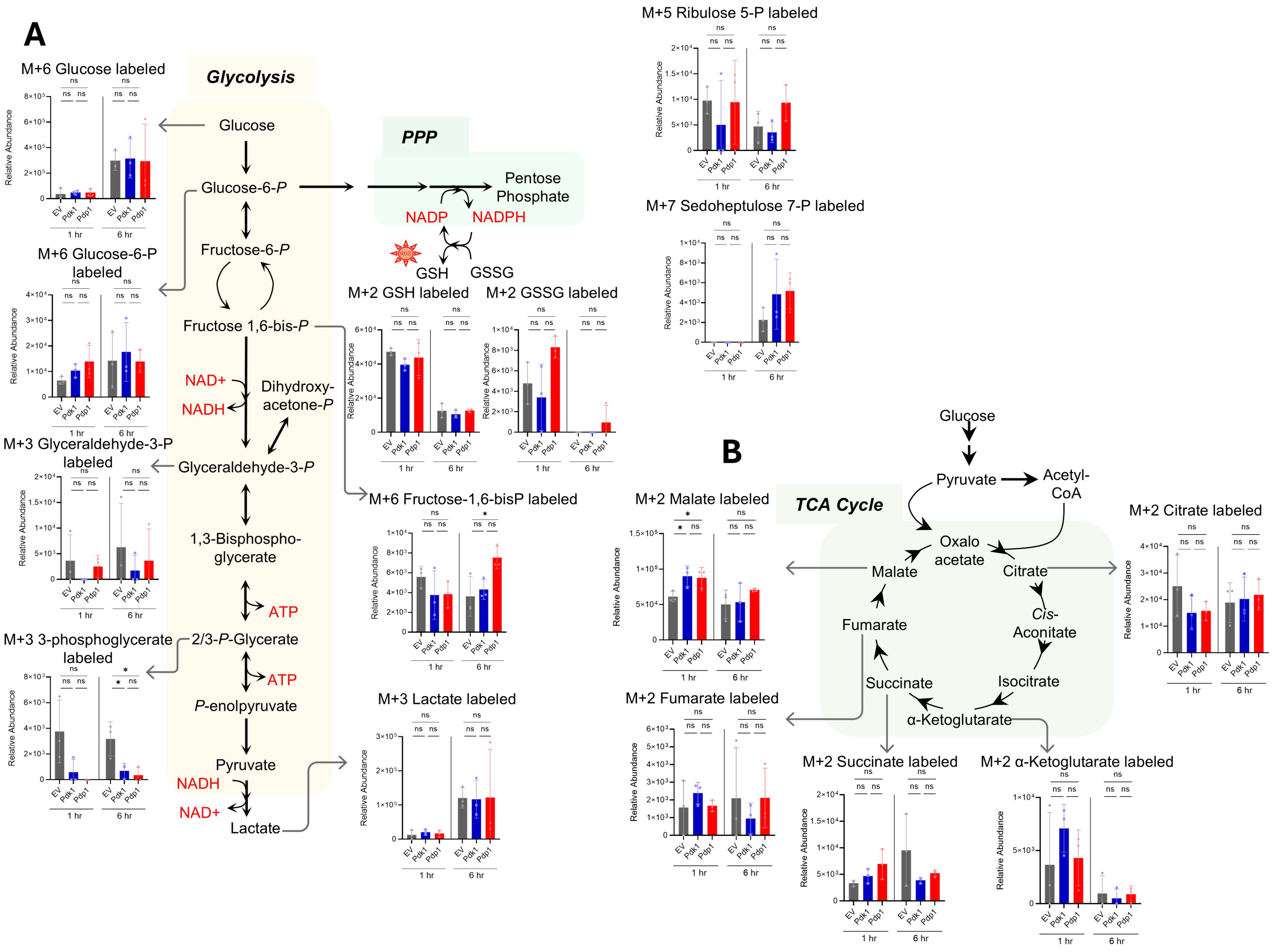

Supplemental Figure 4. ^{13}C glucose tracing reveals similar utilization of glucose in all three groups Wild-type B6 CD8$^+$ T cells transduced with empty vector (EV), PDK1, or PDP1 were cultured with ^{13}C glucose for 1 or 6 hours as in Figure 4-5. Detected metabolites in the **(A)** glycolysis and pentose phosphate pathways and **(B)** TCA cycle pathways are shown per group. n=3 technical replicates per group; One-way ANOVA corrected for multiple comparisons with Tukey's multiple comparisons test. *p<0.05; ns, not significant. Data are mean ± SD.

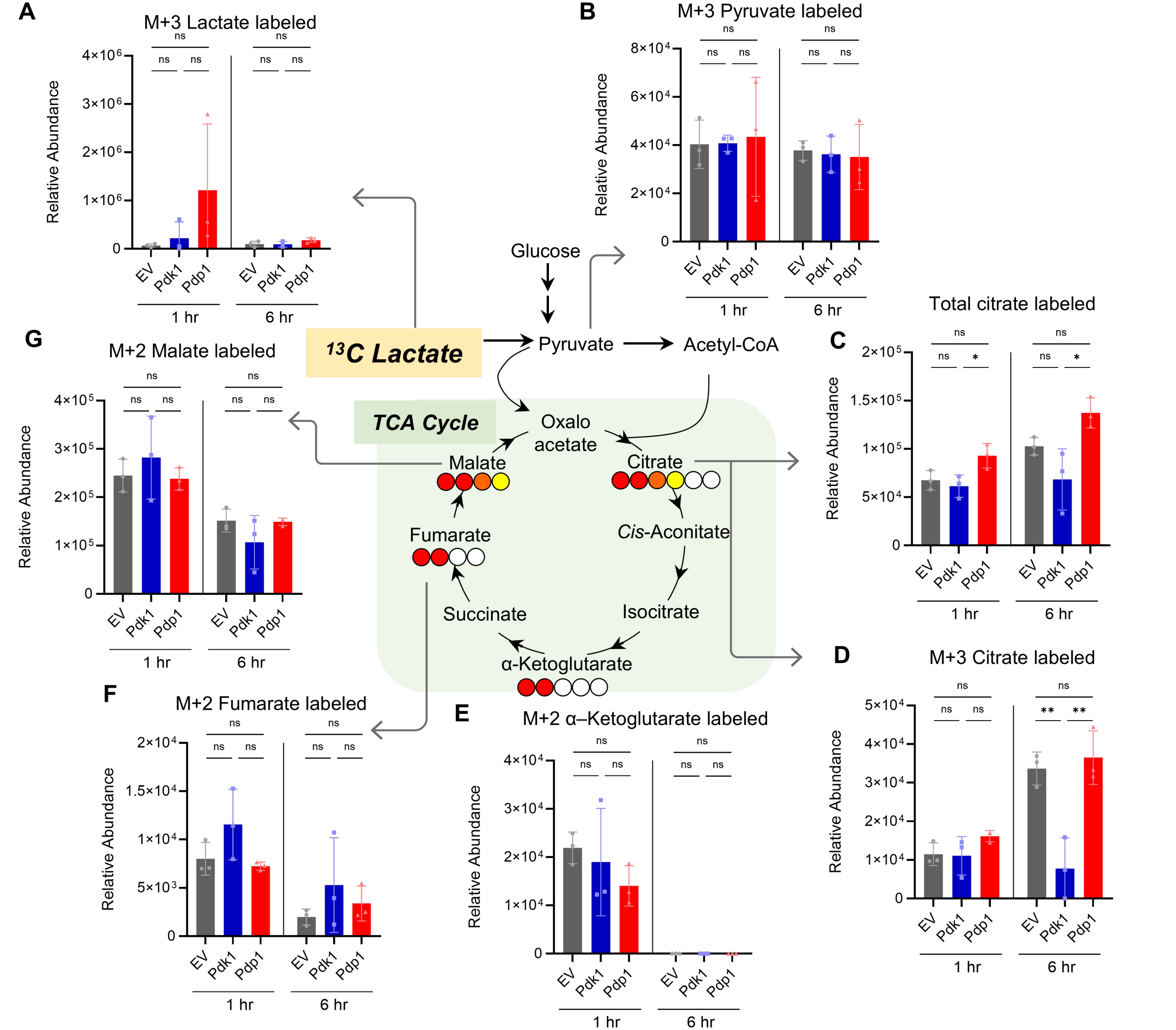

Supplemental Figure 5. ^{13}C lactate tracing reveals slightly increased citrate labeling in the PDP1 group Engineered CD8^{+} T cells with ^{13}C lactate incubation after 1 or 6 hours as in Figure 4-5. TCA intermediates were detected. **(A)** Labeled M+3 lactate uptake and **(B)** labeled M+3 pyruvate. (**C)** Total (M+2, 3, 4) labeled and **(D)** M+3 citrate enriched in the Pdp1 group. Downstream TCA intermediates **(E)** M+2 α-ketoglutarate, **(F)** M+2 fumarate, and **(G)** M+2 labeled. n=3 technical replicates per group; One-way ANOVA corrected for multiple comparisons with Tukey's multiple comparisons test. *p<0.05; **p<0.01; ns, not significant. Data are mean ± SD.

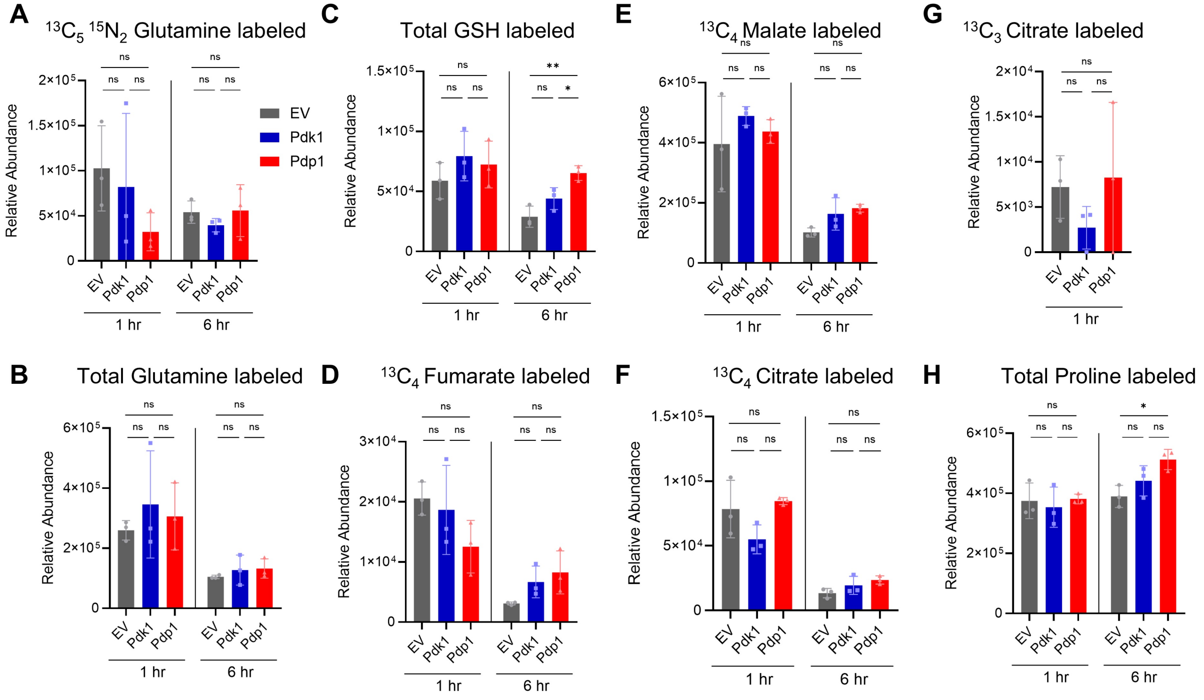

Supplemental Figure 6. $^{13}C^{15}N$ glutamine-derived isotopologues with different labeling suggest a similar trend of altered glutamine utilization in the PDK1 and PDP1 groups Engineered CD8+ T cells with $^{13}C^{15}N$ glutamine incubation after 1 or 6 hours as in Figure 4. **(A)** $^{13}C_5{}^{15}N_2$ labeled glutamine. **(B)** Total labeled glutamate. **(C)** Total labeled GSH. **(D-G)** Downstream TCA intermediates **(D)** $^{13}C_4$ fumarate, **(E)** $^{13}C_4$ malate, **(F)** $^{13}C_4$ citrate, and **(G)** $^{13}C_3$ citrate. **(H)** Total labeled proline. n=3 technical replicates per group; One-way ANOVA corrected for multiple comparisons with Tukey's multiple comparisons test. *$p<0.05$; **$p<0.01$; ns, not significant. Data are mean ± SD.

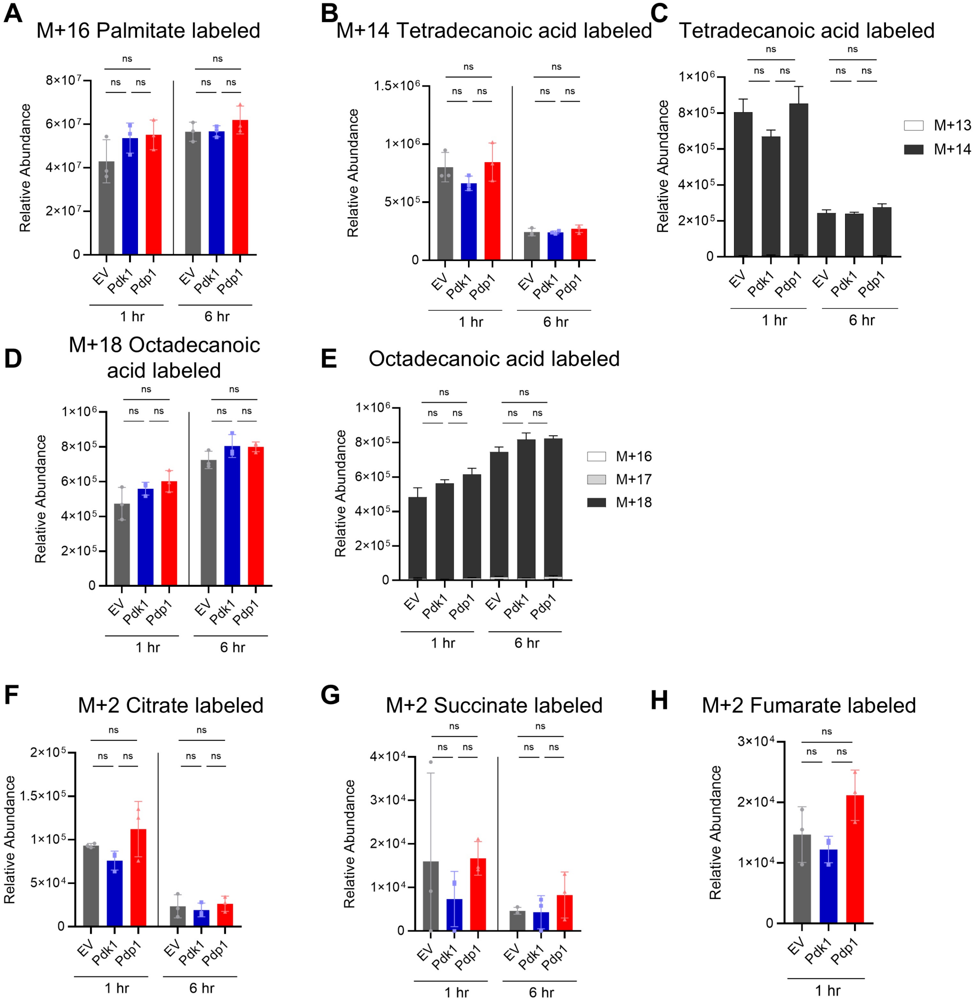

Supplemental Figure 7. ^{13}C palmitate-derived isotopologues with different labeling suggest a similar trend of altered palmitate utilization in the PDK1 and PDP1 groups Engineered $CD8^+$ T cells with ^{13}C palmitate incubation after 1 or 6 hours as in Figure 5. **(A)** M+16 palmitate uptake. Fatty acids of different chain lengths labeled with palmitate-derived ^{13}C including **(B)** M+14 tetradecanoic acid, **(C)** total (M+13, 14) labeled tetradecanoic acid, **(D)** M+18 octadecanoic acid, and **(E)** total (M+16-18) octadecanoic acid. **(F-H)** TCA intermediates labeled with ^{13}C enriched in the PDP1 group, including **(F)** M+2 citrate, **(G)** M+2 succinate, and **(H)** M+2 fumarate (not detected at 6-hour). n=3 technical replicates per group; One-way ANOVA corrected for multiple comparisons with Tukey's multiple comparisons test. *p<0.05; ns, not significant. Data are mean ± SD.

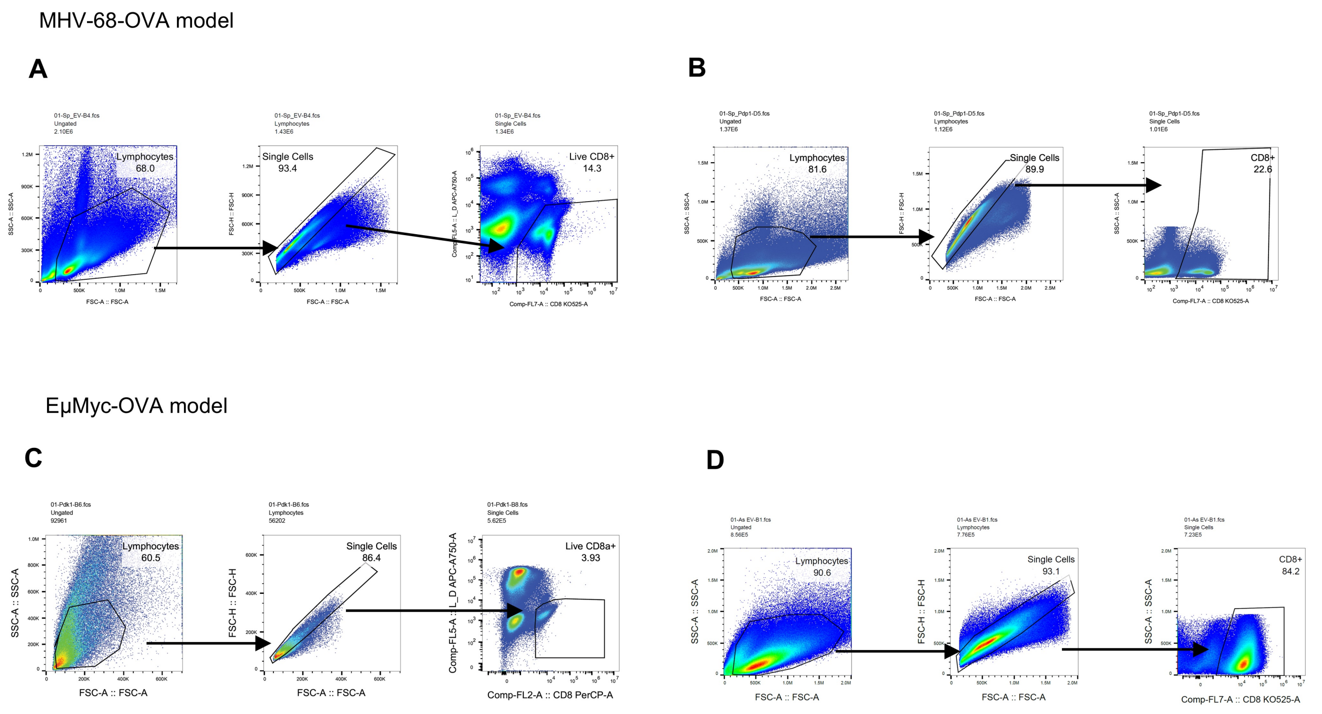

Supplemental Figure 8. Gating strategies for flow cytometry of in vivo adoptively transferred CD8+ T cells in MHV-68 and B cell lymphoma models The transferred OT-I CD8^{+} T cells from MHV-68 and B cell lymphoma mouse models were analyzed by flow cytometry as described in Figures 6-8. The gating strategies for analyzing CD45.1^{+} OT-I^{+} cells in **(A)** the MHV-68-ova model for Ki-67 and cytokine staining and **(B)** Annexin V/7-AAD staining are shown. In addition, gating strategies for CD45.1^{+} OT-I^{+} cells in **(C)** the EμMyc-ova B cell lymphoma model for surface exhaustion markers, Ki-67, and cytokine staining and **(D)** Annexin V/7-AAD staining are shown.

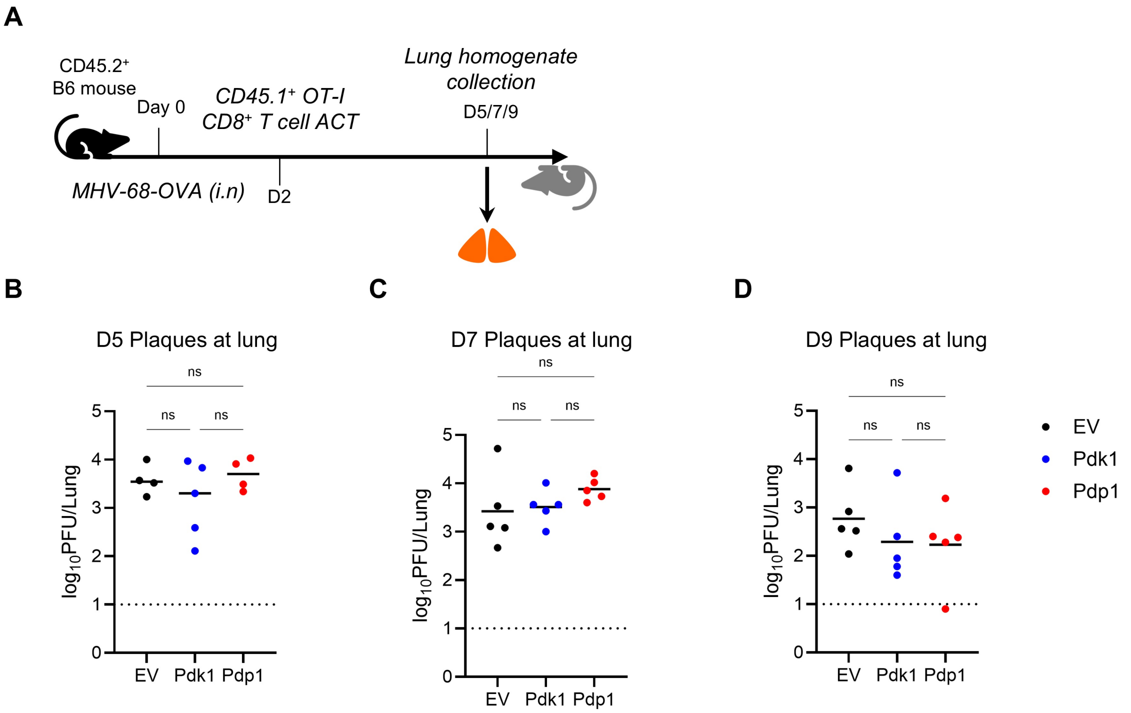

Supplemental Figure 9. Viral loads in MHV-68-OVA mice adoptively transferred with engineered OT-I cells (A) Wild-type CD45.1+ OT-I T cells were retrovirally transduced by the empty vector (EV), PDK1, or PDP1 before adoptive transfer into CD45.2+ C57BL/6 mice infected by murine MHV-68 expressing OVA 2 days before adoptive cell transfer (ACT). **(B-D)** 5, 7, and 9 days post-infection, lungs were harvested to measure viral titers from the tissue homogenates. MHV-68 viral plaques were counted on NIH-3T3 cell monolayers in vitro to compare viral replication between groups. Experiments were repeated 2 times. Dotted lines indicate the limit of detection. n=4-5 biologically independent samples per group; One-way ANOVA corrected for multiple comparisons with Tukey's multiple comparisons test. *p<0.05; ns, not significant.

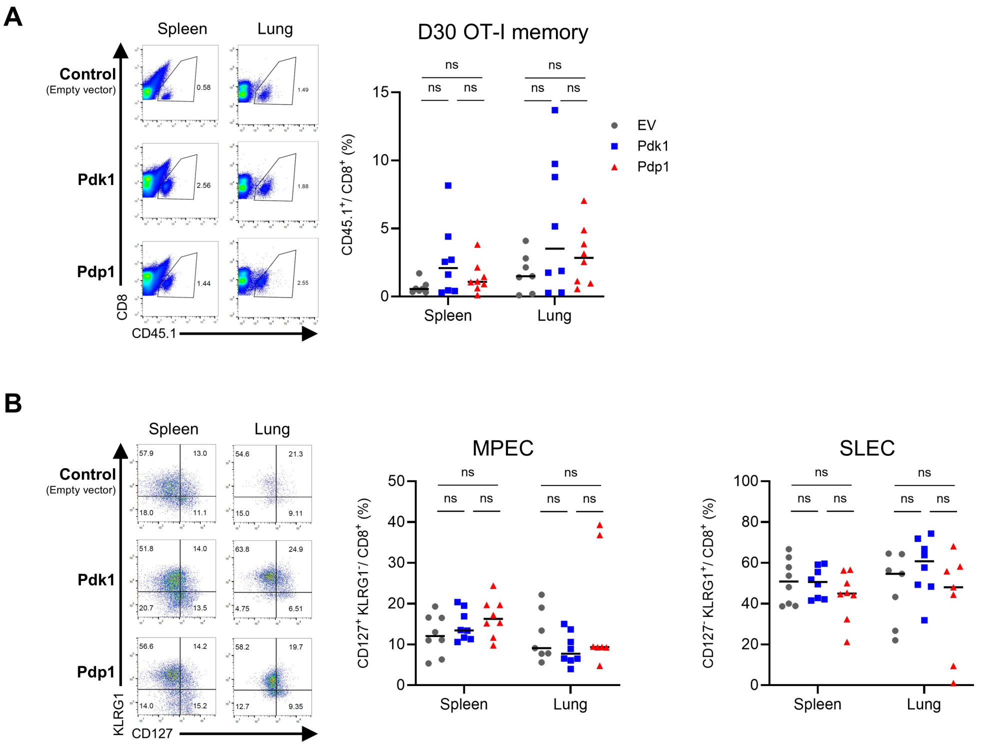

Supplemental Figure 10. Memory precursor effector cells and resting memory cells were not differentially represented in PDK1 and PDP1 groups after MHV-68 infection Wild-type $CD45.1^+$ OT-I T cells isolated from mouse spleens were retrovirally transduced by the empty vector (EV), PDK1, or PDP1 before adoptive transfer into $CD45.2^+$ C57BL/6 mice infected by murine gammaherpesvirus-68 (MHV-68) expressing OVA 4 days prior. **(A)** 30 days later, resting memory $CD45.1^+$ OT-I cells were analyzed by flow cytometry. **(B)** 10 days after adoptive transfer, primary effector $CD45.1^+$ OT-I cells from spleens and lungs were characterized as memory precursor effector cells (MPEC) and short-lived effector cells (SLEC) by flow cytometry. Corresponding flow cytometry plots and dot plots are shown. Experiments were repeated 3 times. n=7-8 biologically independent samples per group; One-way ANOVA corrected for multiple comparisons with Tukey's multiple comparisons test. *p<0.05; ns, not significant.

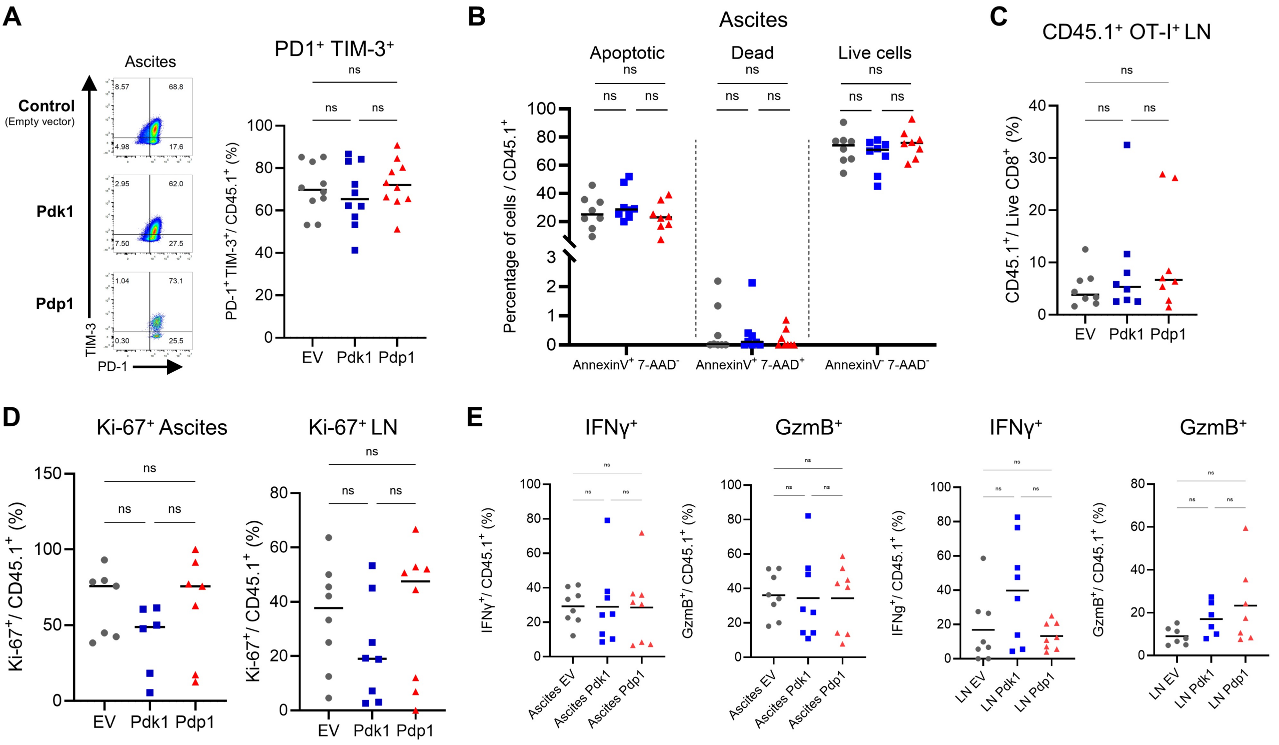

Supplemental Figure 11. Exhaustion, proliferation, and cytokine production of CD8⁺ T cells overexpressing PDH regulatory enzymes in the B cell lymphoma mouse model In the same way as the experimental model described in Figure 8, 1 million EμMyc-ova tumor cells were injected into B6 mice, followed by 500,000 transduced OT-I CD8⁺ T cells 4 days later. **(A)** Exhaustion markers and **(B)** Annexin V/7-AAD staining from ascites, **(C)** OT-I population from inguinal lymph node, **(D)** Ki-67 in ascites and inguinal lymph node, and **(E)** cytokine production in cells isolated from ascites. Experiments were repeated 2-3 times. n=6-8 biologically independent samples per group; One-way ANOVA corrected for multiple comparisons with Tukey's multiple comparisons test. *$p<0.05$; ns, not significant. Data are mean ± SD.

Supplemental Methods

Cloning and generation of DNA constructs

Mouse *Pdk1* (cDNA clone MGC:28719) and *Pdp1* transcript variant 4 (NCBI reference sequence: NM_001290387.1) cDNA sequences from the NCBI sequence database were used for molecular cloning. *Pdk1* and *Pdp1* cDNA clones were purchased from Dharmacon and Origene, respectively. The following primers were used for cDNA amplification: Pdk1-5' For attcacgcgtccgccaccATGAGGCTGGCAAGGCTGCTGCGGG, Pdk1-3' Rev TAGTCGACCGGTTTAAGAGCTTCGGAATGTGGTCATATCTTTCG, Pdp1- 5' For attcacgcgtccgccaccATGGAGCGGCGGCGGTGCGCCTGTC, Pdp1-3' Rev tagtcgACCGGTttaCTGTTCCTGGTTTTGGTATGCC. DNA amplicons were amplified with Phusion HF Taq polymerase (ThermoFisher Scientific, F-530XL) and were digested with the plasmid vector by restriction enzymes BshTI and MluI (Anza). pCI-CD19 plasmid vector modified from pCIGAR retroviral vector by the addition of truncated mouse CD19 was used for the retroviral vector (1).

Metabolomics

$CD8^+$ T cells were isolated from female B6 mice spleens and then stimulated with anti-CD3/28 antibodies for two days. Activated cells were retrovirally transduced and purified using anti-CD19 APC antibody with MojoSort nanobeads (BioLegend, 480072) as described. Cells in the culture were expanded for 5 days. 1 million cells with three technical replicates per condition were cultured in 1 mL of media supplemented by unlabeled or carbon/nitrogen labeled fuel source. XF RPMI Seahorse media (Agilent Technologies, 103576-100) was supplemented with 4.5 mM glucose (Sigma, G7021-1KG), 0.5 mM glutamine (Corning, 25-005-CI), 0.5 mM

palmitate (Sigma-Aldrich, P0500), and 2 mM L-lactate (Sigma-Aldrich L7022) as the unlabeled media. For the specific labeled media, [$^{13}C_6$]glucose (Sigma-Aldrich, 389374-100MG), [$^{13}C_5$, $^{15}N_2$]glutamine (Sigma-Aldrich, 607983-100MG), [$^{13}C_{16}$]palmitic acid (Aldrich, 605573-100MG), or [$^{13}C_3$]Sodium L-lactate (Cambridge Isotope Laboratories, CLM-1579-PK) were added in place of the unlabeled fuel. Palmitic acid was reconstituted in ethanol at 30 mg/mL and further solubilized with 0.083 mM BSA and 6.28 mM NaCl in the final media. Cells were incubated in each labeled media for 1 or 6 hours at 37°C. After incubation, cells were washed three times with cold PBS and the cell pellets were harvested, frozen, and shipped to the University of Colorado on dry ice.

Metabolites from cells were extracted from $2x10^6$ cells per mL at 4°C (30 min) in ice-cold 5:3:2 MeOH:MeCN:water (v/v/v). The samples were spun down at 18,213 g for 10 minutes at 4°C. The resulting supernatant was analyzed on a Thermo Vanquish UHPLC coupled to a Thermo Q Exactive MS as previously described in detail (2). Peak annotation and integration were performed with El-Maven (Elucidata), the KEGG database, and an in-house standard library. Peak areas of $^{13}C_2$-enriched metabolites were corrected for natural abundance. Stable isotope tracing data was visualized using GraphPad Prism 10.0.

Transcriptomics analysis

$CD8^+$ T cells isolated from B6 female mouse spleens were stimulated and transduced. The purified transduced cells were cultured and harvested 3 days later. 500,000 cells with four technical replicates were harvested per group, and the cell pellets were frozen until analysis by bulk RNA sequencing.

RNA was extracted using the RNeasy Mini Kit (Qiagen), quantified by Qubit (Thermo Fisher), and integrity was measured on a Fragment Analyzer instrument (Agilent). 3' RNA-seq libraries

were prepared from 100ng total RNA using the Quantseq FWD v2 workflow (Lexogen) following the manufacturer's instructions. Libraries were pooled for sequencing on a NextSeq2000 instrument targeting 10M, single-end 100bp reads per sample.

FastQC (v0.11.8) was used to assess the quality of raw sequence data before downstream analysis. Single-end reads were subjected to base quality trimming using Cutadapt (v4.0). CollectRNASeqMetrics (Picard Tools,v 2.27.1) was used to assess the quality of read alignments. Gene-level read counts were generated featureCounts (Subread package, v2.0.1) (3) using Ensembl gene build version 98 for GRCm38 and the strand argument set to "1". Raw read counts were analyzed using R-package DESeq2 (4). To evaluate relationships between samples, read counts were transformed using the regularized logarithm method implemented in DESeq2 and analyzed with principal components analysis (PCA) using R function prcomp() and unsupervised hierarchical clustering (using R-package ComplexHeatmap (5)) using the 500 most variable genes. For differential expression (DE) testing, raw read counts were assumed to follow a negative binomial distribution, with empty vector samples used as the reference group. Gene-wise dispersion estimates were reviewed to confirm the negative binomial model was an appropriate fit for the data. Genes with a Benjamini-Hochberg adjusted P-value < 0.05 (Wald-test) and log2 fold change >0.5 were used for overrepresentation analysis (ORA) to assess functional enrichment of significant differentially expressed genes (DEGs). Molecular signature Database (MSigDb) collection C5 (ontology gene sets) were used as the input gene sets to test DEGs for functional enrichment. ORA was performed with function enrichGO, from the clusterProfiler R-package (6), with 'minGSSize' set to 5, 'qvalueCutoff' set to 0.05, and the 'universe' set as all genes tested in the DE analysis. Molecular Function gene sets were also tested, however, few reached statistical significance.

Antibody staining and flow cytometry

Cells were washed twice, FcR-blocked, and stained for LIVE/DEAD stain (LIVE/DEAD Fixable Near-IR Dead Cell stain, Invitrogen L10119) for 15-20 minutes at 4°C in the dark. Cells were then washed twice before surface staining. Samples were stained with antibodies for surface markers in a staining buffer (PBS with 2% bovine growth serum) for 30 minutes at 4°C in the dark. Antibodies used for staining are listed in Supplemental Data 3.

Apoptosis and cell death markers were stained using PE Annexin V Apoptosis Detection Kit with 7-AAD (BioLegend, 640934) following the manufacturer's protocol. Cells were immediately analyzed using a CytoFLEX S (Beckman Coulter) flow cytometer after staining and resuspension in Annexin V binding buffer.

After surface staining, samples were washed twice. Some samples were permeabilized in fixation/permeabilization buffer and further stained for intracellular cytokines.

Cells for intracellular cytokine staining were incubated with 1μg/mL of SIINFEKL peptide in 200 μL T cell culture media with 10μg/mL brefeldin A (Alfa Aesar, J62340) in 96 well U-bottom plates for 5 hours. Brefeldin A was added throughout the staining procedure until the cells were stained for surface antibodies and fixed with paraformaldehyde.

Ki-67 was stained after fixation/permeabilization with pre-chilled 70% ethanol for 1 hour at -20°C. After two washes with PBS, cells were resuspended with Ki-67 antibody diluted 1:100 in staining buffer. After 1 hour of incubation in the dark at room temperature, cells were washed twice and analyzed using a flow cytometer.

Western blot and mitochondrial fractionation

Cells were harvested after 5 to 20 days from in vitro culture with 25 U/mL of rhIL-2. Cell lysates were prepared from 1 million cultured B6 or OT-I $CD8^+$ T cells per sample. The cells were lysed

with 100 µL RIPA buffer (ThermoFisher, 89900) with protease/phosphatase inhibitor (Thermo Scientific, 1861281) followed by two rounds of sonication for 30 seconds each time with a 30-second interval. Cells were mixed with Laemmli sample buffer (Bio-Rad, 1610747), and SDS-PAGE protein electrophoresis was performed. Proteins were transferred to Immobilon®-FL PVDF Membranes (EMD Millipore IPFL00010) and blocked with blocking solution (Li-COR, 927-60001) for 1 hour. Rabbit primary antibodies for PDK1, PDP1, PDH phospho-ser300, and mouse actin (Supplemental Data 3) were incubated with membranes for 16 hours. Membranes were incubated with secondary antibodies IRDye 800CW anti-rabbit (Li-COR, 827-08365) and IRDye 680RD anti-mouse (926-68170) for 1 hour before final washing and detection with an Odyssey CLx Digital Imager (Li-COR) for 680nm (mouse) and 800nm (rabbit) conjugated fluorescent antibodies. Western blot images were analyzed using Image Studio Lite Ver 5.2 (LI-COR) software.

For organelle-specific protein expression analysis, 10 million cells per group were harvested, and nuclear, mitochondrial, and cytosolic fractions were separated as previously described (7). Briefly, cells were resuspended in the filtered extraction buffer (PBS with 220 mM mannitol, 68 mM sucrose, 10 mM Tris-HCl, 50 mM KCl, 2mM $MgCl_2$, and cOmplete™ protease inhibitor cocktail tablet (Roche, 11697498001)), and lysed using a dounce homogenizer with at least 40 strokes per sample. After microscopic confirmation of cell lysis, the nuclear pellets were collected by centrifugation at 1300g for 5 minutes. The supernatant was further separated by centrifugation at 14,000g for 15 minutes for the mitochondrial pellets and cytosolic supernatant. Separated fractions were normalized for protein concentration by Bradford assay. 5 µg of protein per sample was used for SDS-PAGE and the western blot experiment. Rabbit primary antibodies for ATP synthase β and Lamin B1 with other target antibodies described above were used and the western blot procedure was conducted as described for the cell lysate experiment.

Plaque assay and viral load detection

C57BL/6 female mice were intranasally infected by MHV-68-ova with 4,000 U per mouse. Two days later, 20,000 engineered OT-I cells were adoptively transferred into the mice by retro-orbital injection.

For plaque assay, 5, 7, and 9 days after infection, the lungs were harvested and ground by homogenizer. Samples were kept on ice during the procedure. Tissues were homogenized with glass bead for 1 minute, followed by 10-second intervals and another 1 minute of shaking. After bead-beater homogenization, samples were sonicated to release the virus using a Branson sonifier with the same cycle as the homogenizer cycle. Each lung homogenate was resuspended in 1 mL culture media. 100,000 NIH/3T3 cells were plated on 12-well plates per well to form a monolayer. Lung homogenates were serially diluted with culture media, and 500 μL of homogenate was added per well to the NIH/3T3 cell monolayer. 2x supplemented DMEM (Sigma-Aldrich, D7777) media containing 20% newborn calf serum was mixed with an equal volume of carboxymethylcellulose (CMC) (Sigma-Aldrich, C9481) for the rest of the plaque assay media. 2 mL of this media was carefully added to the cell monolayer and incubated at 37°C. After 5 days, cell monolayers were washed with methanol and stained with 8% Giemsa stain solution diluted in water for 5 hours. After staining, the monolayer was washed in running water and dried before plaques were counted to measure the viral titers.

1. Huang YH, et al. Positive Regulation of Itk PH Domain Function by Soluble IP_4. *Science*. 2007;316(5826):886–889.

2. Nemkov T, et al. High-Throughput Metabolomics: Isocratic and Gradient Mass Spectrometry-Based Methods. *Methods Mol Biol Clifton NJ*. 2019;1978:13–26.

3. Anders S, Pyl PT, Huber W. HTSeq--a Python framework to work with high-throughput sequencing data. *Bioinforma Oxf Engl*. 2015;31(2):166–169.

4. Gu Z, Eils R, Schlesner M. Complex heatmaps reveal patterns and correlations in multidimensional genomic data. *Bioinformatics*. 2016;32(18):2847–2849.

5. Love MI, Huber W, Anders S. Moderated estimation of fold change and dispersion for RNA-seq data with DESeq2. *Genome Biol*. 2014;15(12):550.

6. Yu G, et al. clusterProfiler: an R Package for Comparing Biological Themes Among Gene Clusters. *OMICS J Integr Biol*. 2012;16(5):284–287.

7. Bossy-Wetzel E. Mitochondrial cytochrome c release in apoptosis occurs upstream of DEVD-specific caspase activation and independently of mitochondrial transmembrane depolarization. *EMBO J*. 1998;17(1):37–49.
