# Supplementary material for: Targeting pyruvate metabolism generates distinct CD8+ T cell responses to gammaherpesvirus and B lymphoma
Source: JCI Insight. 2025 Aug 22;10(16):e187680. doi: 10.1172/jci.insight.187680 (PMC12406717; doi:10.1172/jci.insight.187680)

Full unedited gels for Figure 1B

PDK1

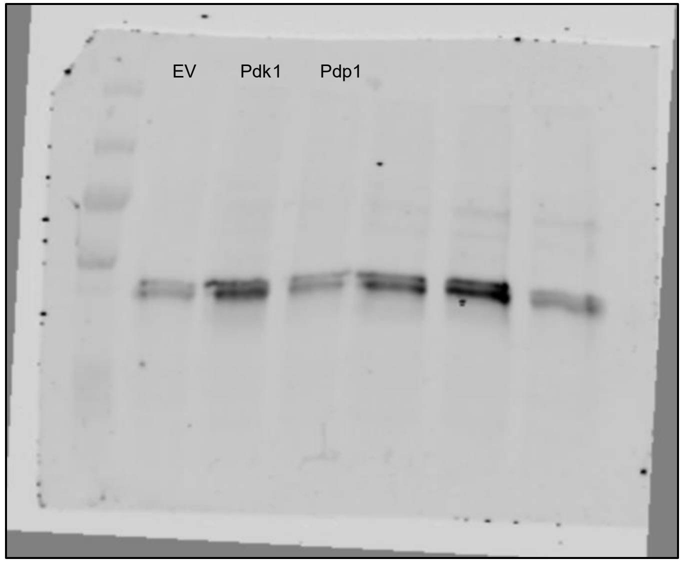

PDP1

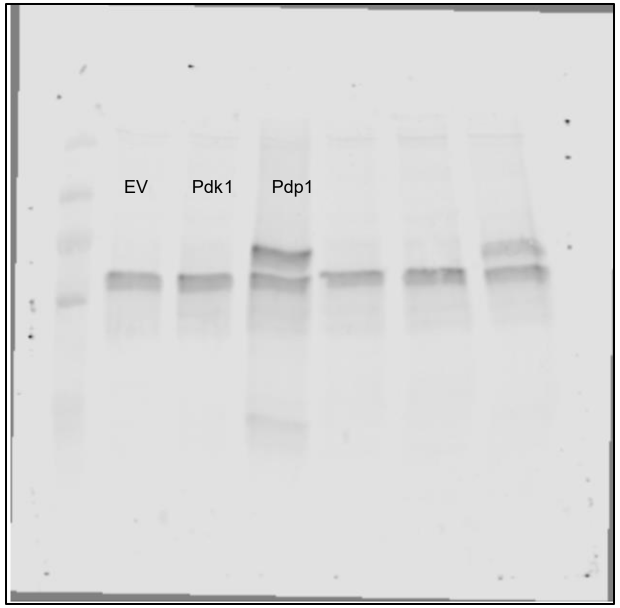

pPDH

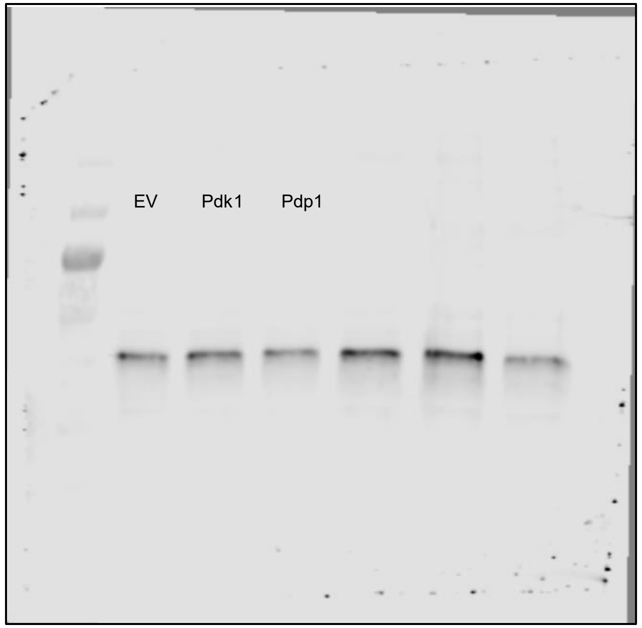

Actin

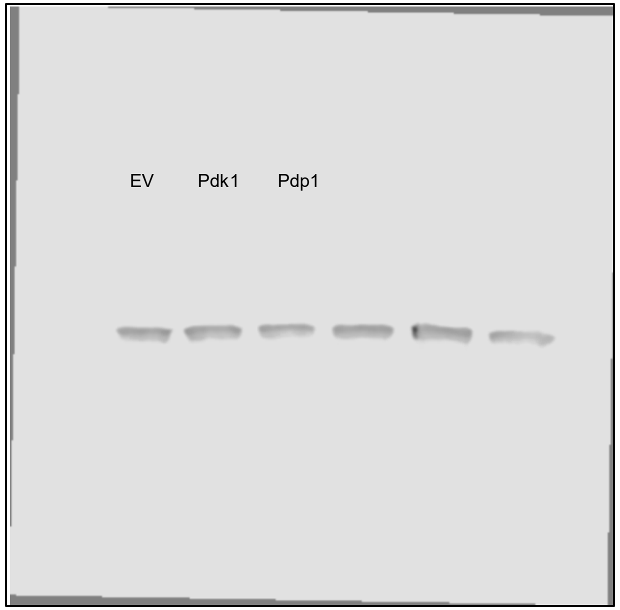

Full unedited gels for Figure 1C

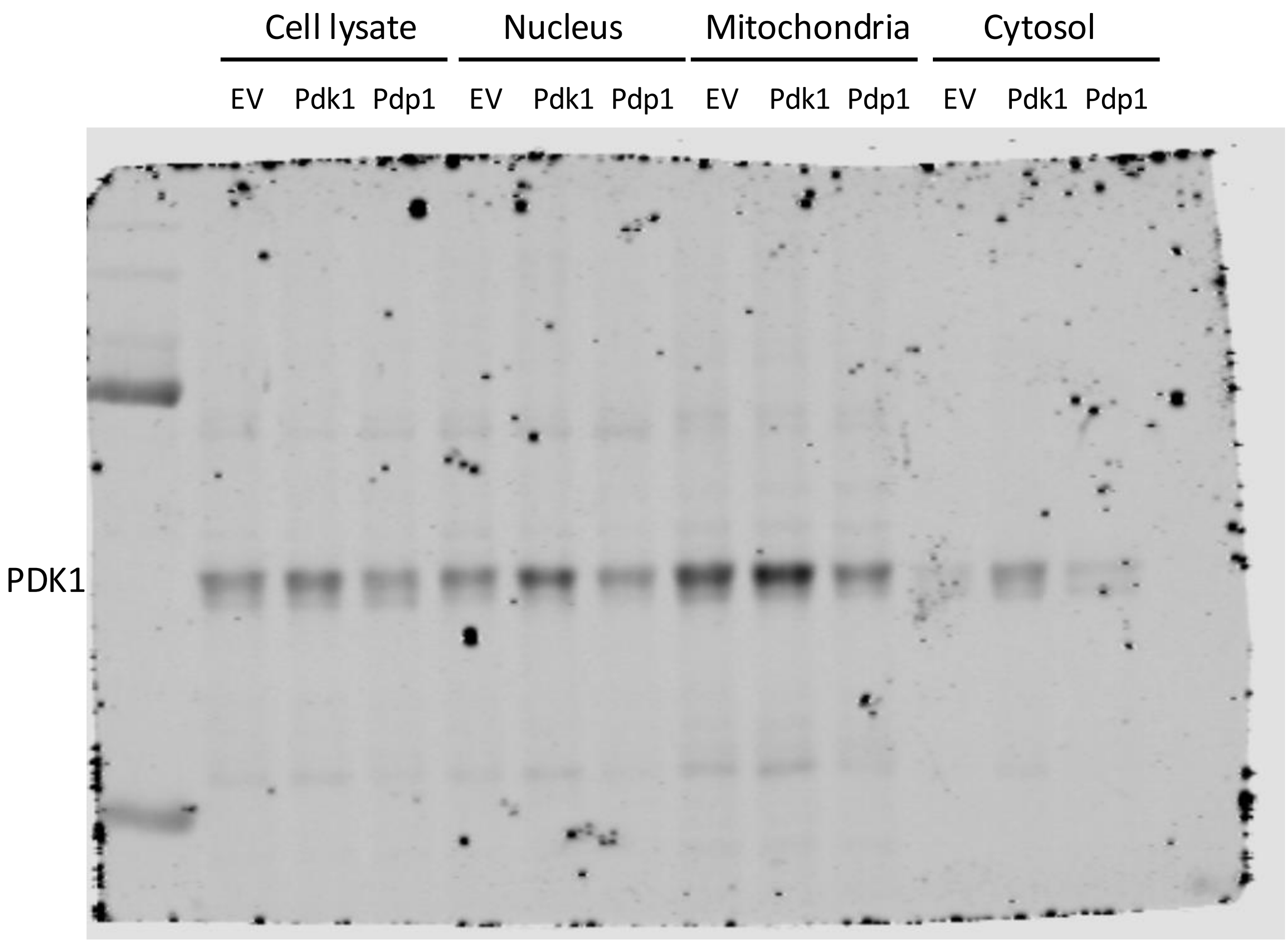

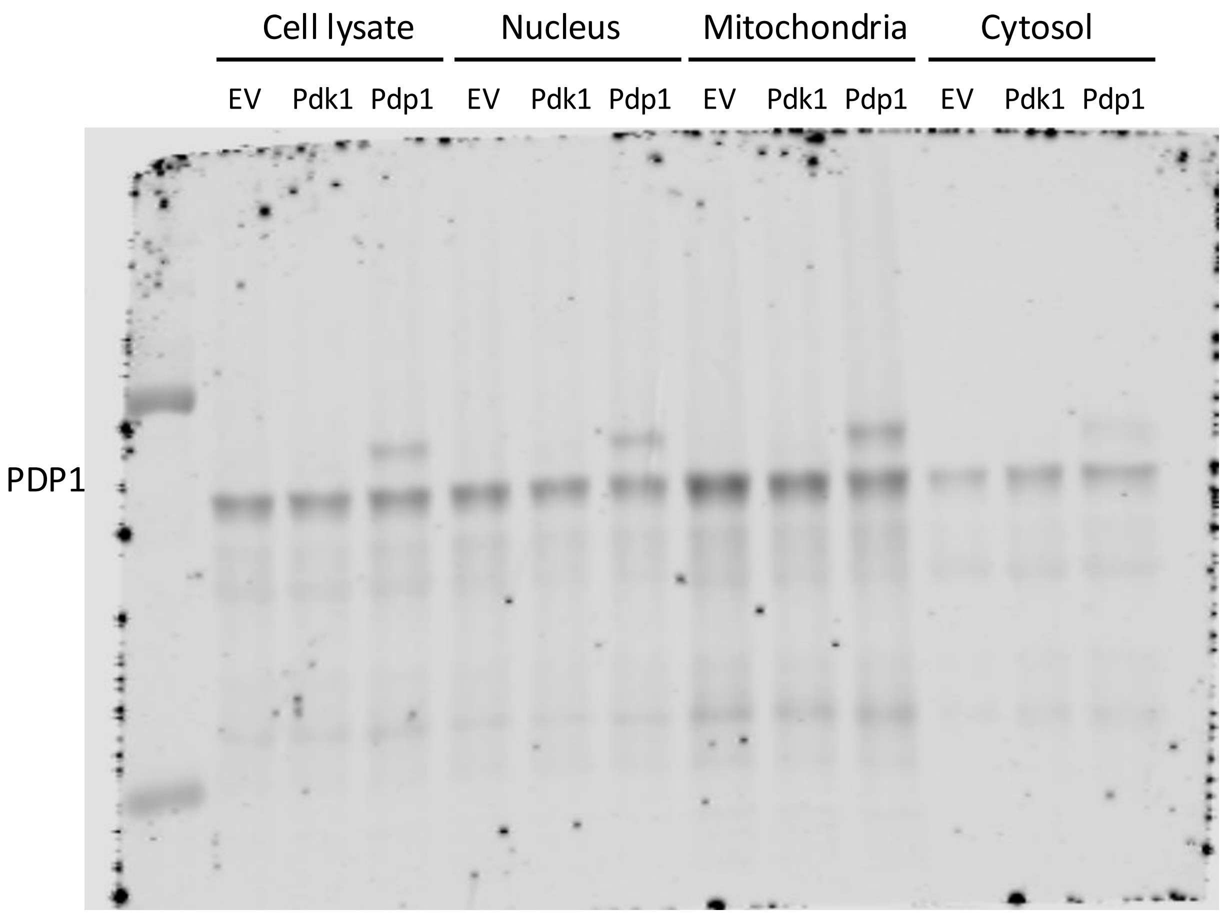

Full unedited gels for Figure 1C

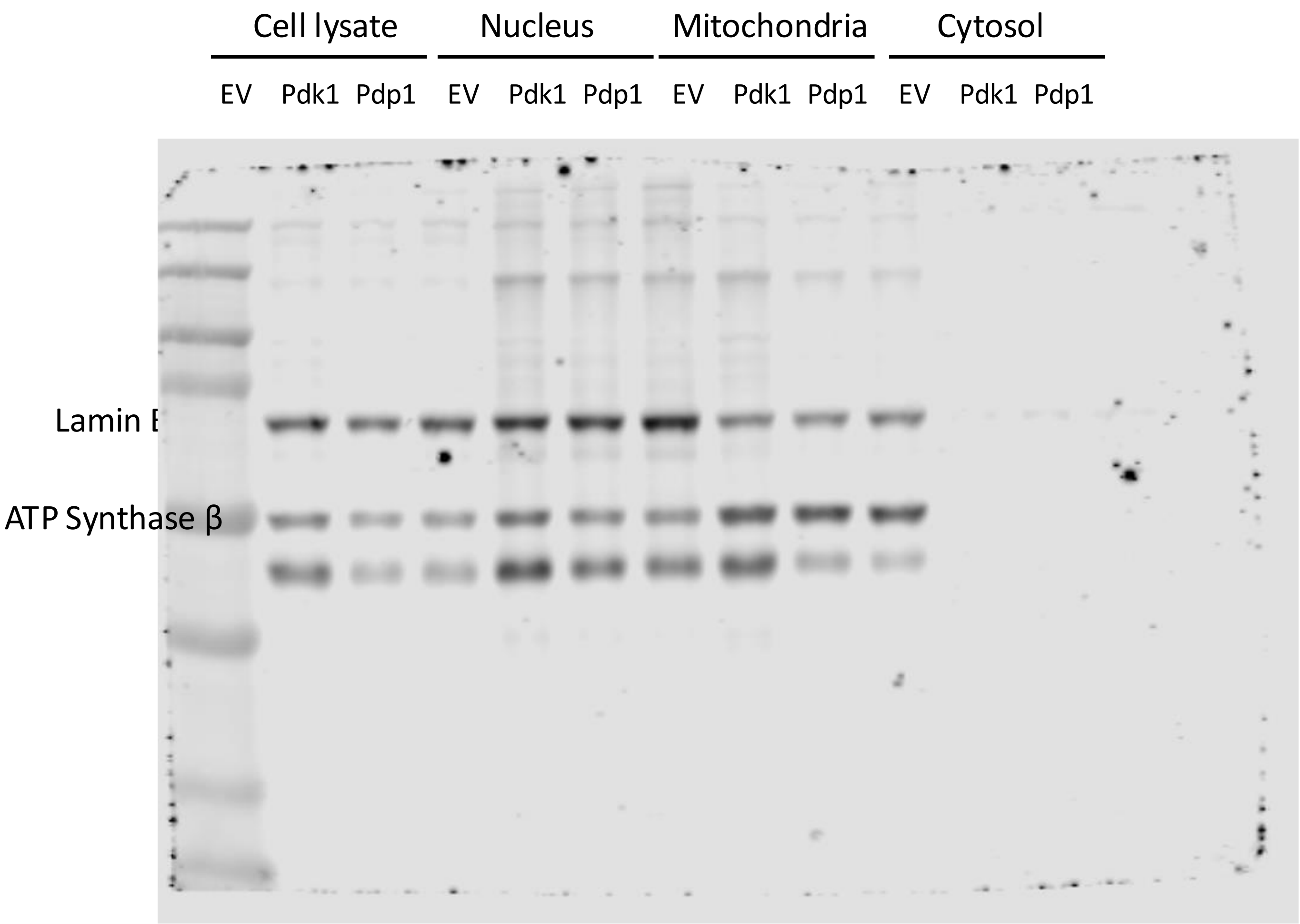

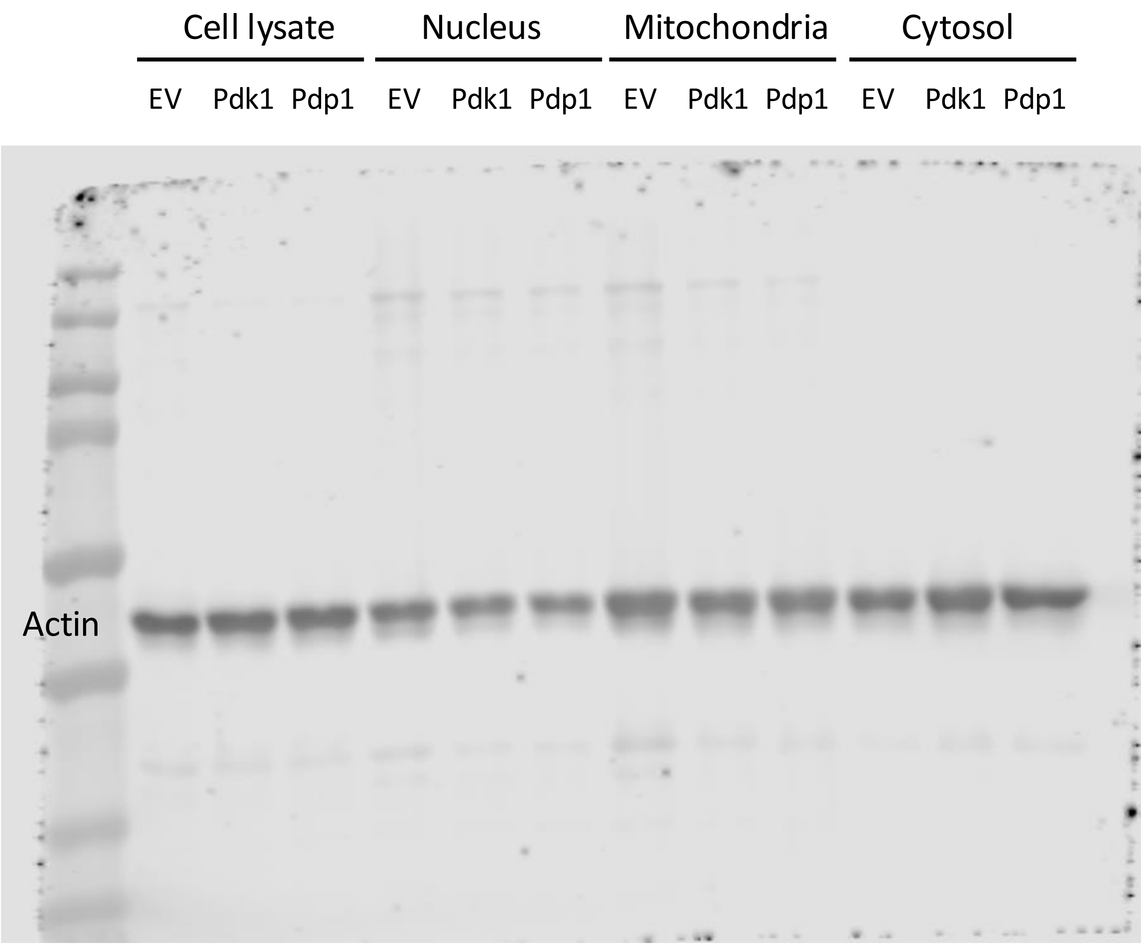

Full unedited gels for Figure 1D

Mitochondria

EV Pdk1 Pdp1

pPDH

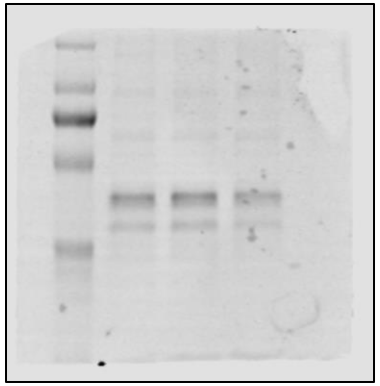

Mitochondria

EV Pdk1 Pdp1

ATP Synthase β

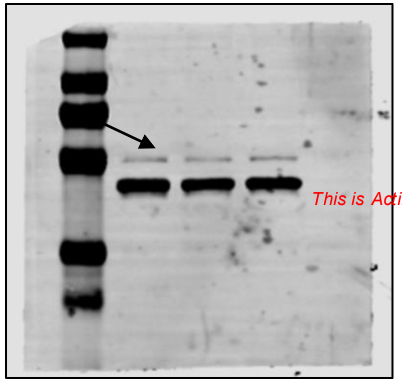

This is Actin

Full unedited gels for Supplemental Figure 2E

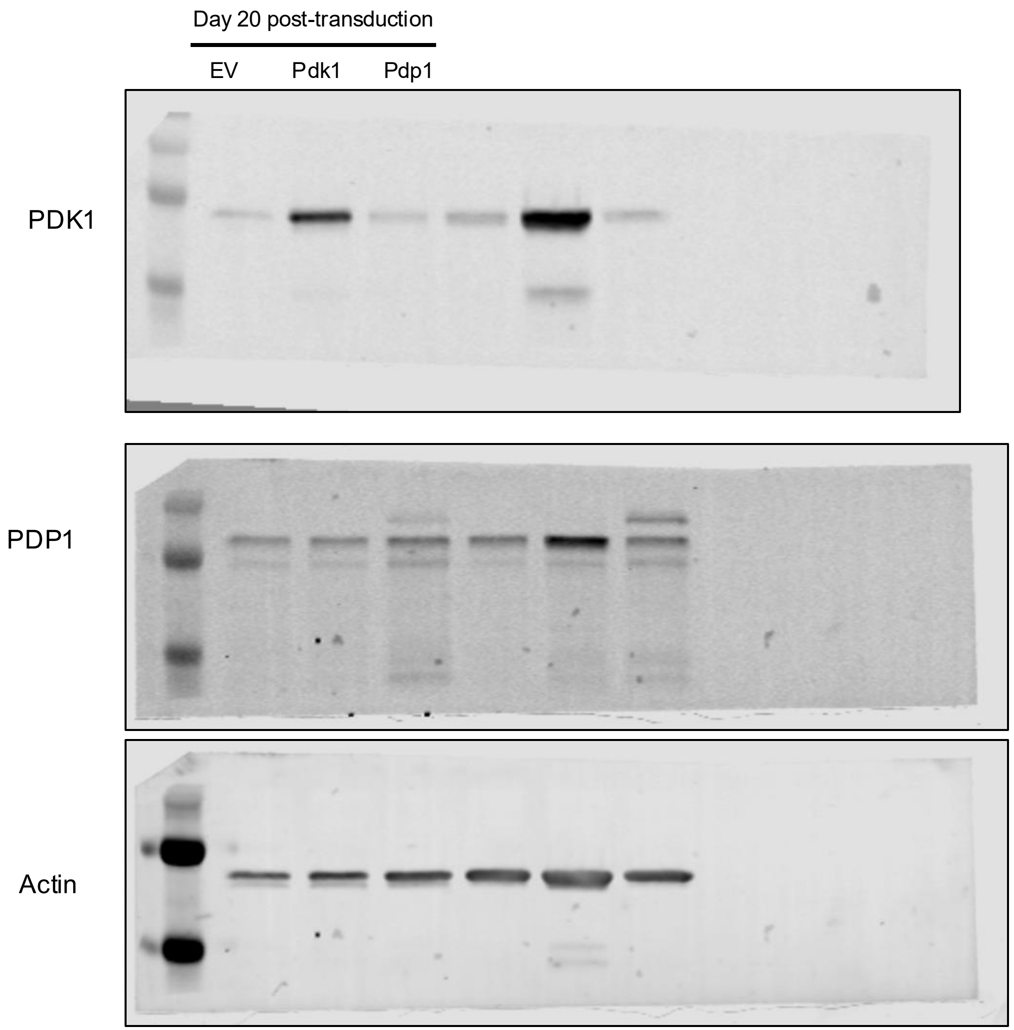

Supplement: Unedited blot and gel images [file jciinsight-10-187680-s297.pdf]
